# Spin State in Au Porphyrins Modulated by Charge Transfer on Au(111)

**DOI:** 10.1021/jacs.5c21710

**Published:** 2026-03-24

**Authors:** Donglin Li, Manish Kumar, Oleksandr Stetsovych, Benjamin Lowe, Rima Sengupta, Hironobu Hayashi, Hiromitsu Maeda, Pavel Jelínek, Shigeki Kawai

**Affiliations:** † Center for Basic Research on Materials, 52747 National Institute for Materials Science, 1-2-1 Segen, Tsukuba, Ibaraki 305-0047, Japan; ‡ Institute of Physics, 86889 Academy of Sciences of the Czech Republic, Cukrovarnicka 10, Prague 6 CZ 16200, Czech Republic; § Department of Condensed Matter Physics, Faculty of Mathematics and Physics, Charles University, Prague 2 CZ 12116, Czech Republic; ∥ Department of Applied Chemistry, College of Life Sciences, 29491 Ritsumeikan University, Kusatsu 525-8577, Japan; ⊥ Graduate School of Pure and Applied Sciences, University of Tsukuba, Tsukuba, Ibaraki 305-8571, Japan

## Abstract

Controlling spin states at the single-molecule level is a crucial step toward functional molecular spintronic devices. Au porphyrins, as efficient electron acceptors, are highly sensitive to charge transfer on surfaces and offer a promising route to investigate spin-state modulation in single-molecule magnets. Here, we report the synthesis of phenalenyl-expanded Au porphyrins via cyclodehydrogenation on Au(111). The atomic-scale structures, electronic properties, and spin states of the products were investigated in detail with a combination of noncontact atomic force microscopy, scanning tunneling microscopy, scanning tunneling spectroscopy, as well as density functional theory and multireference quantum chemistry calculations. Although the structures are nearly identical, the spin states of the porphyrins are significantly affected by the charge state of the Au complex. Our findings show that the role of the molecule–substrate interactions and the resulting charge transfer of the gold complex tune the spin and electronic properties of the extended porphyrins, establishing them as versatile molecular platforms for investigating charge-transfer-driven spin switches and guiding the design of molecular spintronic devices.

## Graphical abstract

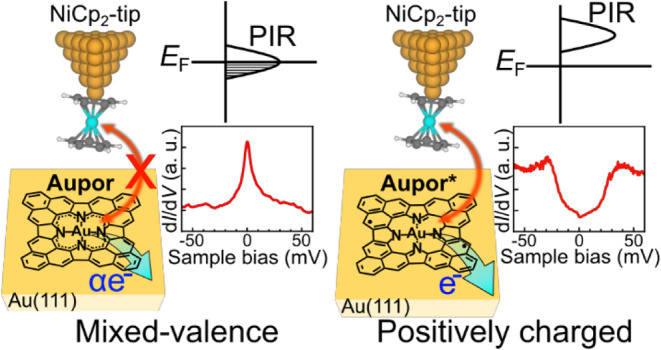

## Introduction

Single-molecule magnets (SMMs) are molecular systems that behave as individual magnetic units, characterized by large spin ground states and pronounced magnetic anisotropy.^1–3^ Their atomic-scale spin degrees of freedom and long coherence times make them promising candidates for high-density information storage,^4,5^ spintronics,^6–8^ and quantum computing.^9–12^ Among them, metalloporphyrins represent a prototypical class of SMMs that have attracted particular attention because their electronic and magnetic properties can be finely tuned through variation of the central metal ion.^13–15^ This versatility allows precise control over charge transport, spin configuration, and magnetic anisotropy, positioning metalloporphyrins as ideal molecular platforms for probing fundamental aspects of molecular electronics and spintronics. Consequently, achieving atomic precision in their structural engineering and systematically investigating their magnetic and electronic properties at the single-molecule level are essential steps toward realizing functional spintronic devices.

On-surface synthesis has emerged as a powerful bottom-up strategy for constructing atomically precise open-shell spin architectures.^16–21^ Under ultrahigh vacuum conditions, rational precursor design combined with controlled surface-assisted reactions has enabled the creation of a variety of open-shell nanographenes, such as triangulenes,^22,23^ butterfly-shaped nanographenes,^24^ and Clar goblet structures.^25,26^ Extending this approach to porphyrin frameworks, phenalenyl-expanded porphyrins have been synthesized. Their spin states and magnetic anisotropy are modulated by the presence or absence of a central metal, as well as by the number and arrangement of phenalenyl units.^27–31^ These studies demonstrate the potential of porphyrin scaffolds for tailoring molecular magnetism via π-extension. However, despite these advances, the influence of strong charge-transfer abilities associated with the central metal on the magnetic properties of expanded porphyrins remains largely unexplored. Unraveling these effects not only deepens our fundamental understanding of molecular magnetism but also provides design principles for engineering advanced molecular electronic and spintronic devices.

Here, we focus on phenalenyl-expanded Au-centered porphyrins (Au porphyrins), fabricated via on-surface cyclodehydrogenation of 5,10,15,20-tetrakis­(2,6-dimethylphenyl) precursors on Au(111) (Scheme 1). The Au porphyrin complexes have been reported to exhibit strong charge-transfer characteristics,^32,33^ providing an excellent platform for investigating metal–ligand and molecule–substrate interactions. The chemical structures of the products were identified using noncontact atomic force microscopy (nc-AFM). Scanning tunneling microscopy (STM) and scanning tunneling spectroscopy (STS) revealed two distinct classes of Au porphyrins with different spin states, highlighting the sensitivity of their magnetic properties to subtle variations in charge transfer. The experimental observations are rationalized by density functional theory (DFT), multireference complete active space (CAS) calculations, and a many-body model Hamiltonian, revealing the important role of charge transfer in determining the spin ground state of Au porphyrins. Together, these results establish Au porphyrins as versatile molecular platforms for investigating charge-transfer-driven spin phenomena.

**Scheme 1:**
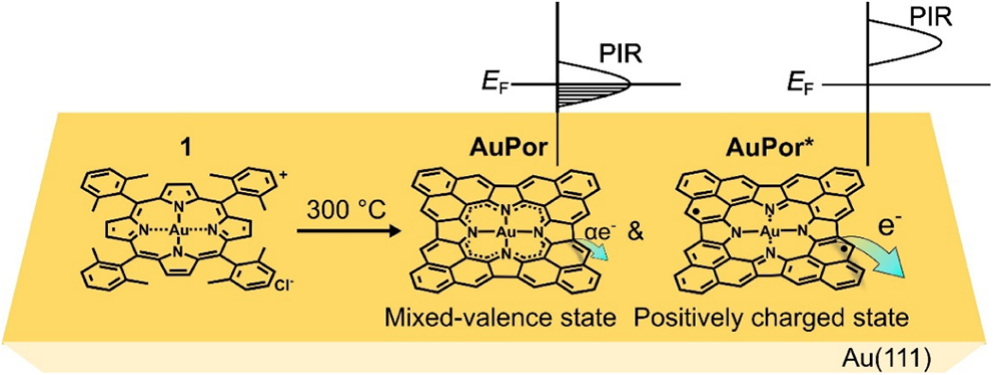
Synthetic Route of Phenalenyl Expanded Au Porphyrins Using 5,10,15,20-Tetrakis­(2,6-dimethylphenyl)­porphyrin Au Complex (**1**) as a Precursor and Distinct Alignment of Positive Ionic Resonance (PIR) of the Au Porphyrin Products with Respect to the Fermi Level (E_F_) of the Au(111) Surface^a^

a The different PIR alignment causes a distinct charge state of the Au porphyrin products. Pinning of PIR to the Fermi level gives rise to the **AuPor** product in the mixed-valence regime with a partial charge transfer αe-, where α < 1, while the charge transfer of one electron e- results in the AuPor* product in the open-shell singlet ground state.

## Results and Discussion

Upon depositing precursor **1** on the Au(111) surface, followed by annealing at 300 °C, surface-assisted cyclodehydrogenation occurred between the methyl groups and the macrocycle, yielding numerous isolated products, as shown in Figure S1a. Careful inspection allowed us to identify that some of the isolated products had a *D*_4_-symmetric structure, as expected for the Au porphyrins shown in Scheme 1 (red boxes in Figure S1a). Other products either feature five-membered carbon rings, which are typical byproducts of cyclodehydrogenation of methyl groups, or lack a central Au atom, likely due to the high annealing temperature (see Figure S1). Herein, we focus on the characterization of the Au porphyrin products. Notably, all isolated Au porphyrins adopt only three orientations, as shown in Figure S2, with a relative rotation of 30° between them. This orientational preference is most likely due to alignment with the underlying Au(111) lattice.

While surveying the surface, we observed subtle differences in the apparent STM contrast of some Au porphyrin products on the surface. As shown in Figure 1a and e, STM images of two *D*_4_-symmetric molecules had a slightly different appearance, with the molecule in Figure 1a exhibiting strong molecular orbital nodes, while the molecule in Figure 1e has more homogeneous intensity across the molecule with stronger intensity at the center, although the spatial distribution is similar. To distinguish between these two products, we will refer to the molecule shown in Figure 1a as **AuPor** and the molecule in Figure 1e as AuPor*. To understand the difference between the two molecules, we performed constant-height ncAFM measurements with a CO-functionalized tip to resolve their chemical structures. As shown in Figure 1b and f, these images clearly reveal a very similar chemical structure of the Au porphyrins, featuring symmetric protrusions at the corners characteristic of phenalenyl units.^34^ Strikingly, these two images appear identical, suggesting that there is no major difference in the chemical structures of **AuPor** and AuPor*. This is further supported by Δ*f*(Δ*z*) measurements performed on the two molecules, which suggest an equivalent adsorption height upon the surface (see Figure S3). In contrast, a clear difference between the two products is evident in the simultaneously acquired STM images in Figure 1c and g. **AuPor** has intensity distributed across the molecule, whereas AuPor* has much stronger intensity at its center than across the rest of the molecule. The equivalent appearance of **AuPor** and AuPor* in ncAFM images and the inequivalence in STM images suggest the molecules have identical chemical structures but different electronic properties. To compare the electronic properties of **AuPor** and AuPor*, we performed d*I*/d*V* STS measurements (Figure 1d and h) on both molecules at the sites of the red dot markers in Figure 1a,e. Near-Fermi STS measurements of the **AuPor** molecule revealed a zero-bias peak, possibly reminiscent of a Kondo resonance. By contrast, near-Fermi STS measurements of the AuPor* molecule exhibit two symmetric steps at ±27 mV, suggestive of inelastic excitation. Notably, among ∼55 intact Au porphyrins identified from STM surveys of ∼550 molecules, 16 **AuPor** and 14 AuPor* molecules were carefully characterized, revealing an approximately 1:1 ratio between the two species (Figure S4).

**Figure 1:**
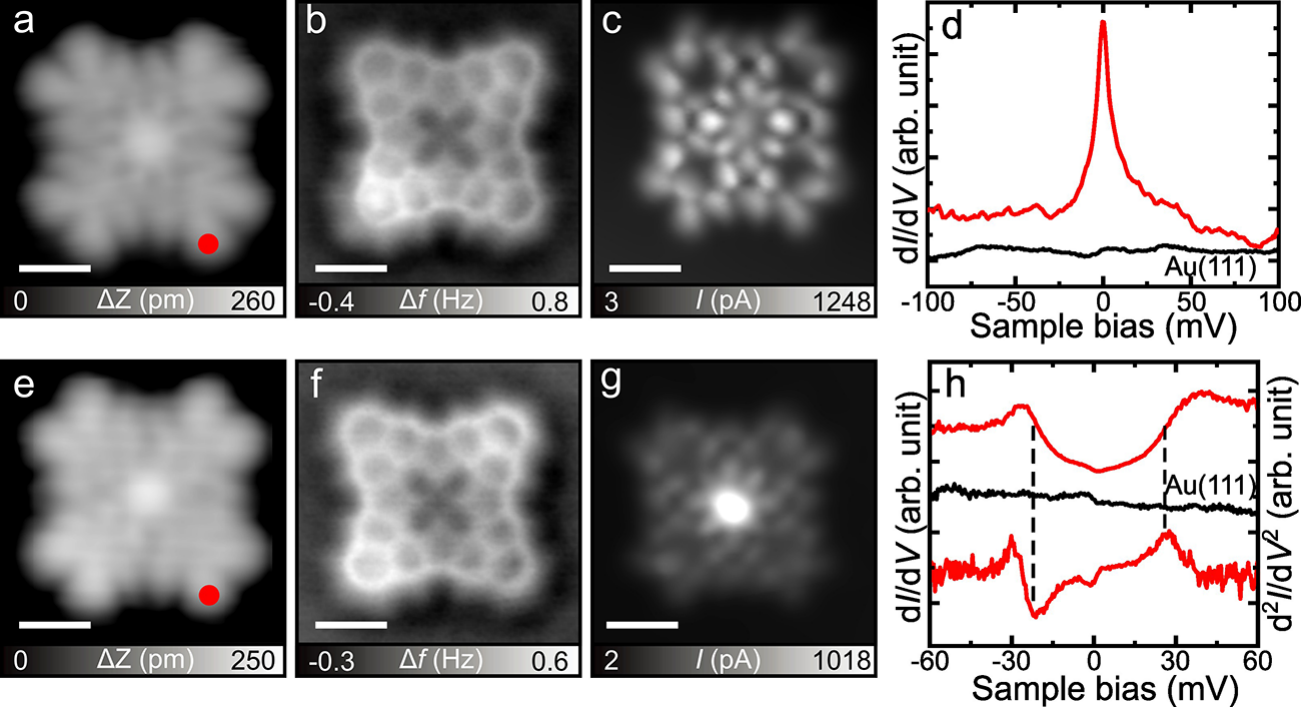
Characterization of two types of Au porphyrins with CO-functionalized tips. (a) Close-up constant-current STM image of **AuPor**. (b, c) Simultaneously acquired constant-height nc-AFM and STM images of **AuPor**, respectively. (d) d*I*/d*V* spectrum taken over the marked site in (a). (e) Close-up constant-current STM image of AuPor*. (f, g) Simultaneously acquired constant-height nc-AFM and STM images of AuPor*, respectively. (h) d*I*/d*V* spectrum and d^2^ *I*/d*V* ^2^ spectrum taken over the marked site in (e). d*I*/d*V* curves in black were taken over the bare Au(111) surface. Scanning parameters: (a) *V* = 0.04 V, *I* = 5 pA; (e) *V* = 0.03 V, *I* = 10 pA; (b, c, f, g) *V* = 1 mV; (d, h) lock-in zero-to-peak modulation voltage *V*_mod_ = 1 mV. Scale bars: 5 Å.

Further electronic characterization of the **AuPor** and AuPor* molecules over a larger energy range also revealed subtle differences between the molecules. For **AuPor**, we found four pronounced peaks at −1.6 V, −0.2 V, 0.6 V, and 1.9 V in the spectra measured at the sites indicated by blue and red dots in the STM image (Figure 2a). The d*I*/d*V* maps acquired at these energies (Figure 2b) reveal the spatial distributions of the states, which are predominantly localized at the edges at positive bias, while they are also localized at the center at negative bias. The long-range d*I*/d*V* spectra measured at the same sites of AuPor* (Figure 2d) exhibit subtly different peaks, as one state observed at negative bias at the center of **AuPor** is absent. Although no apparent peak was detected, we found another state at −0.8 V through careful inspection of a series of d*I*/d*V* maps recorded in the range of −2.0 V to 1.6 V (Figure S5).

**Figure 2:**
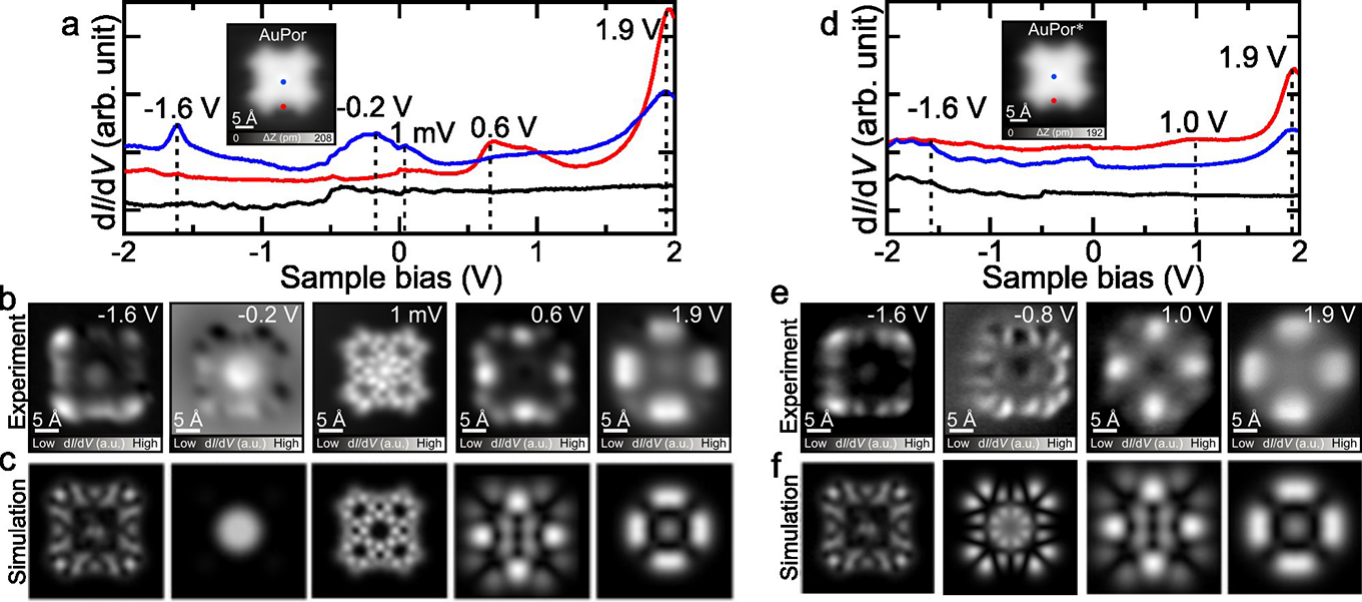
Electronic properties of Au porphyrins. (a) Long-range d*I*/d*V* spectra of **AuPor** taken over the marked dots in the inset STM image. d*I*/d*V* curve in black was taken over the bare Au(111) surface. *V_mod_* = 10 mV. (b) Constant-current d*I*/d*V* maps of **AuPor** taken at −1.6, –0.2, 0.001, 0.6, and 1.9 V with a CO tip. (c) Simulated d*I*/d*V* maps of the Dyson orbitals of **AuPor**. (d) Long-range d*I*/d*V* spectra of AuPor* taken over the marked dots in the inset STM image. d*I*/d*V* curve in black was taken over the bare Au(111) surface. *V_mod_* = 10 mV. (e) Constant-current d*I*/d*V* maps of AuPor* taken at −1.6, −0.8, 1.0, and 1.9 V with a CO tip. (f) Simulated d*I*/d*V* maps of the Dyson orbitals of AuPor*. Scanning parameters: (a, d) *V* = 0.2 V, *I* = 10 pA; (b, e) *I* = 100 pA, *V*_mod_ = 10 mV.

Motivated by these experimental observations, we performed a multilevel theoretical study combining single-determinant total energy DFT slab calculations, multireference complete active space configuration interaction (CASCI) calculations, and many-body model Hamiltonian methods to gain more insight into the electronic and magnetic structure of the distinct species **AuPor** and AuPor*, respectively.

First, we examined the electronic structure of the product in its neutral form using DFT and CASCI calculations in the gas phase. Both computational methods predicted an open-shell doublet ground state, characterized by a single unpaired electron residing in a localized singly occupied molecular orbital (SOMO), with a significant contribution from the d-orbital of the central Au atom (see Figure S6). The multireference ground-state wave function obtained from CASCI­(11,11) shows a dominant contribution from one Slater determinant (Figure S7a), which justifies the validity of the single-determinant DFT method in describing the neutral state of the product.

Next, we carried out slab DFT calculations of the neutral product on a three-layer Au(111) slab (Figure S8). The optimized geometry exhibits a large adsorption height of ∼3.35 Å, in good agreement with the experimental data (3.33 Å, Figure S3) obtained from force spectroscopy. Also, analysis of the projected density of states shows negligible hybridization of molecular orbitals with the electronic states of the metallic substrate (Figures S8 and S9), confirming a noncovalent interaction between the molecule and the surface. Importantly, Bader charge analysis indicates a net charge transfer of ∼0.9 e from the molecule to the substrate. In DFT calculations, the charge transfer occurs from the localized SOMO orbital with strong d-like character to the Au(111) surface, which is now pinned to the Fermi level; see Figure S8 below. According to DFT as well as DFT + U (Figure S10) calculations, the charge transfer of one electron from SOMO results in quenching of the magnetic moment and closed-shell character of the molecule; see the symmetric character of the spin-resolved PDOS of the molecule on the surface shown in Figures S8 and S10. This charge transfer is almost independent of the different adsorption positions of the molecule with respect to the surface, as shown in Figure S8. The closed-shell singlet ground state of the positively +1e charged molecule in the gas phase is predicted by DFT calculations using different exchange-correlation functionals (see Methods in Supporting Information).

Moreover, the single-determinant DFT method has certain limitations in accurately describing the electronic structure and charge transfer in organic/metal complexes.^35–38^ Namely, the single-determinant DFT cannot properly describe the inherently multireference mixed-valence regime and the alignment of molecular ionic resonance with the Fermi level of the metal surface.

Nevertheless, based on the possibility of charge transfer from the molecule to the substrate predicted by DFT calculations, we propose a scenario that explains the emergence of two distinct STS spectra for otherwise chemically identical molecules, **AuPor** and AuPor*. This scenario involves the presence of a molecule in two different charge states: the positively charged +1e state (AuPor*) with one electron fully transferred to the surface from the molecule, and the mixed-valence regime (**AuPor**), where the molecular ionic resonance is pinned to the Fermi level but the molecule retains a partially neutral character, as shown in Figure 1.

However, DFT calculations reveal two major discrepancies in the interpretation of experimental data when using this scenario. First, the simulated d*I*/d*V* maps corresponding to the one-electron canonical DFT molecular orbitals for neutral (**AuPor**) and positively charged +1e (AuPor*) molecules do not match well with the experimental d*I*/d*V* maps. In the case of the neutral molecule, the main deficiency is that d*I*/d*V* maps associated with the first ionic resonance, corresponding to the removal of an electron, fail to match the experimental results. The experimental contrast is primarily localized on the ligand, whereas the theoretical d*I*/d*V* map exhibits a strongly localized signal on the metal part, associated with the SOMO possessing a strong d-like character (see Figure S11). In the case of AuPor*, we observed a similar deficiency for the first ionic resonance corresponding to the addition of an electron, where the theoretical d*I*/d*V* maps again correspond to the strongly localized d-like SOMO state, which cannot replicate the experimental contrast (see Figure S12).

Second, in the case of AuPor*, DFT systematically predicts a closed-shell singlet ground state. This observation contradicts the fact that STS spectra of AuPor* exhibit a low-energy spin excitation signal, indicating the magnetic ground state of the molecule. Therefore, in this instance, DFT calculations categorically cannot explain the experimental findings. To verify the failure of the single-determinant DFT method for accurately describing the electronic structure of the +1e charged state, we performed multireference CASCI calculations on the charged molecule in the gas phase. Note that the gas-phase approximation can be justified by the presence of the noncovalent interaction between the molecule and the metallic substrate, with negligible hybridization between the molecular states and the metal, as confirmed both experimentally and theoretically. The CASCI­(12,12) calculations predict the open-shell singlet ground state with a strong multireference character (see Figure S13). Figure S14b displays the CASCI-calculated natural orbitals, which reveal two unpaired electrons in two distinct natural orbitals with different spatial localizations. One unpaired electron is hosted by the strongly localized d-like orbital, which is very similar to the canonical SOMO DFT orbital. The second unpaired electron is located in a natural orbital and is delocalized over the ligand. The distinct electronic character of **AuPor** and AuPor* is also reflected in the unpaired electron density obtained from multireference CASCI calculations, shown in Figure S15. Importantly, CASCI­(12,12)-NEVPT2 ^39^ calculations determine the first excited triplet state at 44 meV above the open-shell singlet ground state. Therefore, this finding naturally explains the inelastic excitation observed in STS measurements of the AuPor* molecule as a magnetic singlet–triplet excitation. Moreover, the simulated d*I*/d*V* maps obtained from natural transition orbitals (NTOs) corresponding to the singlet–triplet transition match very well with the experimental d*I*/d*V* map of the spin excitation (Figure S16),

The experimental d*I*/d*V* maps of ionic resonance of **AuPor** and AuPor*, can be rationalized using the multireference description of STS maps with Dyson orbitals,^40,41^ which include virtual transitions to multireference charged states. This approach, assuming neutral and positively +1e charged molecules, yields very good agreement with the experimental data of **AuPor** and AuPor*, respectively (see Figures 2b–f, S17 and S18). The main difference compared with the canonical one-electron DFT orbitals is that the lowest ionization processes do not take place in a strongly localized *d-*like orbital. This is related, first, to the change in the ground state of the +1e molecule and, second, to the inclusion of correlation effects arising from the Coulombic repulsion between molecular orbitals with different localization. Note that CASCI calculations for the anionic state (Figures S7c, S19) yield electronic structures incompatible with the experimental d*I*/d*V* maps, further supporting the conclusion that the adsorbed species are either neutral or positively charged.

Next, we will address the origin of the zero-bias peak observed for the **AuPor** molecule. In principle, the zero-bias peak in Figure 1d can be tentatively explained as a spin-1/2 Kondo resonance due to the monoradical doublet ground state of the neutral molecule. However, after lateral manipulation of the molecule by the tip (Figure S20), the zero-bias peak shifted to a larger positive bias and became asymmetric. A similar broad and asymmetric peak near the Fermi level was also observed for another **AuPor** molecule prior to manipulation (Figure S21). These findings rule out the presence of Kondo screening. Instead, we attribute this broad peak to a mixed-valence regime, where the resonance near the Fermi level corresponds to the positive ionic resonance of the molecule pinned to the Fermi level of the Au(111) surface, as suggested by the DFT slab calculations (Figure S8). We assume that upon adsorption onto Au(111), the first ionic resonance of **AuPor** shifts from its gas-phase energy and becomes pinned at the Fermi level of the substrate. This pinning is characteristic of a mixed-valence state,^42^ where charge fluctuations quench the molecule’s local spin.

To explore the magnetic properties of the mixed-valence regime, we introduce the many-body model Hamiltonian, which describes charge transfer between a single impurity (molecule) and a finite chain representing a broad metallic band of the surface. The model predicts that when a singly occupied molecular orbital is located near the Fermi level of the surface, the system enters the mixed-valence regime, characterized by strong charge fluctuations between the molecule and the metallic band. In this situation, the charge on the molecular state is no longer an integer, and the spin on the molecule is no longer a good quantum number. For details, see Supporting Information, Section II, Figures S22–S24.

The hypothesis that different charge transfer regimes are the source of the difference between **AuPor** and AuPor* molecules also suggests that the two molecules should exhibit different magnetic properties. In the case of **AuPor**, the molecule should be magnetically silent, as it is in the mixed-valence regime, where strong charge fluctuations between the molecule and the sample quench the magnetic moment. On the other hand, AuPor*, featuring magnetic singlet–triplet excitation, should exhibit a strong magnetic signal. To further test this hypothesis, we performed STS measurements using a NiCp_2_-functionalized tip. Recent work has established NiCp_2_-functionalized tips as magnetic sensors, in which the tip–sample distance-dependent exchange coupling between the NiCp_2_ molecule and a magnetic sample induces characteristic changes in the inelastic excitation spectra.^43–46^ This technique has been shown to be capable of producing signatures that allow for discrimination between different magnetic ground states.^47^ The reliability of the NiCp_2_ probe was first tested by measuring bare Au(111) (Figure S25), where no shift in the characteristic ±4 mV peaks was observed across all tip–sample distances. For **AuPor**, which is in a mixed-valence regime and lacks a pure spin state, no interaction with NiCp_2_ was anticipated (Figure 3a). As anticipated, whether the tip was positioned at the center or the corner (red and blue dots in Figure 3b), no shift in the characteristic NiCp_2_ ± 4 mV peaks was detected at any tip–sample distance (Figure 3c and d). These observations provide further evidence that the zero-bias peak of **AuPor** does not originate from a Kondo screening^46^ but is attributed to the mixed-valence regime.

**Figure 3:**
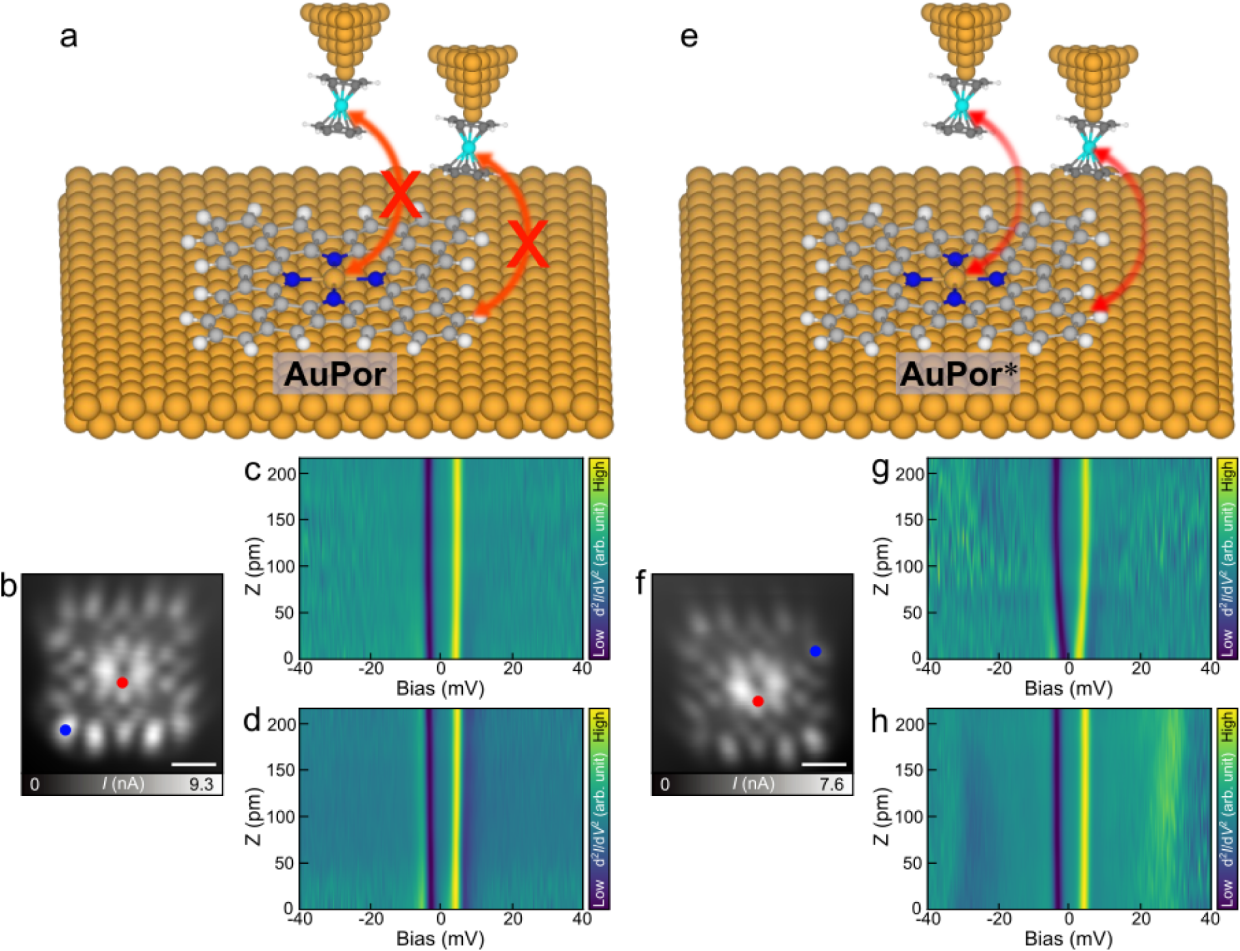
Magnetic characterization of Au porphyrins with a NiCp_2_-functionalized tip. (a) Illustration of the interaction between the NiCp_2_ tip and **AuPor**. (b) STM image of **AuPor** obtained with a NiCp_2_ tip. (c) Height-dependent map composed of a series d^2^ *I*/d*V* ^2^ spectra acquired with a NiCp_2_ tip at the center of **AuPor,** marked by the red dot in (b). (d) Height-dependent map composed of a series d^2^ *I*/d*V* ^2^ spectra acquired with a NiCp_2_ tip at the corner site of **AuPor,** marked by the blue dot in (b). (e) Illustration of the interaction between Nicp_2_ tip and AuPor*. (f) STM image of AuPor* obtained with a NiCp_2_ tip. (g) Height-dependent map composed of a series d^2^ *I*/d*V* ^2^ spectra acquired with a NiCp_2_ tip at the center of AuPor*, marked by the red dot in (f). (h) Height-dependent map composed of a series d^2^ *I*/d*V* ^2^ spectra acquired with a NiCp_2_ tip at the corner site of AuPor*, marked by the blue dot in (f).

In contrast, NiCp_2_ measurements on AuPor* (Figure 3e) revealed distinct magnetic interactions. When the tip was positioned at the molecule center (red dot Figure 3f), the ±4 mV peaks gradually shifted toward the Fermi level with decreasing tip–sample distance (Figure 3g). When the tip was located at a corner site of AuPor* (blue dot in Figure 3f), two sets of peak/dip features were observed: the ±4 mV peaks originating from NiCp_2_ and additional features at ±31 mV arising from coupling between NiCp_2_ and AuPor* (Figure 3h). With a decreasing tip–sample distance, the ±31 mV features broadened and shifted to lower energies.

To understand this behavior, we developed a spin model combining a Heisenberg spin Hamiltonian with cotunneling theory to simulate the corresponding IETS spectra^46,47^ (see Supporting Information, Section II, Figure S26). In the weak-coupling regime, the Heisenberg model predicts three features: a peak at 4 mV (NiCp_2_ excitation), a peak at 27 mV (AuPor* excitation), and a third peak at 31 mV (simultaneous excitation of both NiCp_2_ and AuPor*). However, in the cotunneling-based simulations, only the 4 and 27 mV peaks appear bright; the 31 mV joint excitation is suppressed by cotunneling selection rules, which restrict observable transitions based on spin symmetry and perturbative coupling to the electron reservoir (Figure S26c, d). A schematic energy diagram of the spin excitations probed by IETS is provided in Figure S27. The key to understanding the experimental results lies in the spatial distribution of the molecular spin involved in the spin excitation and its overlap with the NiCp_2_ tip.

When the NiCp_2_ tip is positioned over the molecular center of AuPor* (Figure 3g), it interacts directly with the highly localized spin state of the central Au atom. Simulated constant-height d*I*/d*V* maps of the spin excitation, derived from the natural transition orbitals (NTOs) of the singlet–triplet transition (Figure S16), confirm this pronounced spatial localization at the Au site. This strong, direct overlap between the NiCp_2_ tip and the Au spin results in the 4 mV NiCp_2_ excitation undergoing strong renormalization and shifting toward zero bias as the tip–sample distance decreases, and, in contrast, the weaker 27 mV and 31 mV signals shift gradually toward higher bias (see Figure S26c).

In contrast, when the tip is positioned over a corner site of the molecule (blue dot in Figure 3f), the interaction changes fundamentally. Here, the NiCp_2_ tip couples with the highly delocalized molecular spin in the π-system (Figure S15b). In this weak coupling of NiCp_2_ with the π-spin and Au spin together, the 4 mV NiCp_2_ signal is suppressed. Instead, the dominant effect is a shift of the 27 mV molecular excitation peak toward lower bias as the NiCp_2_ tip approaches the molecule (Figure S26d). The differences in the response of **AuPor** and AuPor* to a NiCp_2_-functionalized probe provide further evidence of a different charge state for the two molecules.

Very good agreement between experimental and theoretical data reveals the delicate balance in charge transfer between the surface and the molecule, which governs the charge state of Au porphyrins. For **AuPor**, pinning of a molecular ionic resonance to the Au(111) Fermi level leads to the mixed valence state in which the molecular spin is quenched. Other molecules lose one electron to the surface and become positively charged, leading to a singlet ground state with antiferromagnetic coupling between a delocalized molecular state on the ligand and a strongly localized *d-*like state on the central Au atom. Different sample preparations yielded slightly different relative abundances of **AuPor** to AuPor*, so we are unable to make any conclusive comment on their stabilities based on yield. In addition, we also examined metal-free porphyrin and Zn porphyrin, both of which exhibit only a single spin state (Figure S28). This observation further supports the conclusion that the Au center plays a crucial role in tuning the spin state of metal porphyrins.

We note that converting one type of porphyrin into another via SPM tip manipulation is very rare. One hypothesis is that the difference in the charge state of the molecules may originate from different adsorption configurations of the molecules, either with respect to the bridge/hollow/top sites of the surface or with respect to the Au(111) herringbone pattern. However, we were able to reproducibly manipulate the molecules laterally on the surface without ever observing a change from **AuPor** to AuPor* or vice versa (see Figure S29), although we did observe small variations in the zero-bias peak of **AuPor** molecules during manipulation experiments (see Figure S20). Additionally, atomic-resolution registration images revealed no obvious difference in the adsorption sites of the two types of molecules on the Au(111) surface (Figure S30).

On the other hand, on rare occasions, we were able to convert AuPor* to **AuPor** via strong interaction with the SPM probe, as shown in Figure S31. Additionally, a detailed analysis of high-resolution nc-AFM images (Figure S32) reveals a small but not negligible variation in the bond lengths of the central part of the molecule between AuPor* an**d AuPor**. Therefore, we tentatively associate the bistability induced by distinct charge states with a distinct geometric relaxation of the porphyrin core that prevents spontaneous or externally stimulated switching.

## Conclusion

In summary, we have successfully synthesized phenalenyl-expanded Au­(III) porphyrins on Au(111) and resolved their chemical structures using nc-AFM and BR-STM. Two distinct charge states of Au porphyrins were identified, exhibiting different electronic and magnetic behaviors due to a subtle variation in charge transfer between the molecule and the substrate. Partial charge transfer sets the molecule in the mixed-valence regime, while one-electron charge transfer causes the open-shell singlet ground state. STS measurements, including NiCp_2_-tip, corroborated with multireference calculations, reveal that only Au porphyrin in the open-shell singlet ground state shows pronounced spin excitations and tip-sensitive magnetic features. These findings highlight the critical role of molecule–substrate interactions and the central metal charge state in modulating molecular magnetism. Our work establishes Au porphyrin as a tunable single-molecule system for studying charge-transfer-driven spin phenomena, providing design principles for the development of advanced molecular electronic and spintronic devices.

## Supplementary Material



## Notes

Methods, including details of STM experiments and theoretical calculations (PDF), additional STM images, orientation analysis, DFT and CASCI calculations, including adsorption site analysis, Bader charge analysis, PBE+U results, Dyson orbitals, manipulation experiments, Single Impurity Anderson Model, spin model for NiCp_2_ tip–molecule interaction, Natural Transition Orbitals, and adsorption registry analysis (PDF)
