# Supplementary material for: Spin State in Au Porphyrins Modulated by Charge Transfer on Au(111)
Source: J Am Chem Soc. 2026 Mar 24;148(13):13863–70. doi: 10.1021/jacs.5c21710 (PMC13067267; doi:10.1021/jacs.5c21710)
Supplement: Supplementary file 1 [file ja5c21710_si_001.pdf]

Spin State in Au-porphyrins Modulated by Charge Transfer on Au(111)

Donglin Li[1‡], Manish Kumar[2,3‡], Oleksandr Stetsovych[2], Benjamin Lowe[2], Rima Sengupta[4], Hironobu Hayashi[1], Hiromitsu Maeda[4], Pavel Jelínek[2]*, Shigeki Kawai[1,5]**

[1]Center for Basic Research on Materials, National Institute for Materials Science, 1-2-1 Segen, Tsukuba, Ibaraki 305-0047, Japan.

[2]Institute of Physics, Academy of Sciences of the Czech Republic, Cukrovarnicka 10, Prague 6, CZ 16200, Czech Republic.

[3]Department of Condensed Matter Physics, Faculty of Mathematics and Physics, Charles University, Prague 2 CZ12116, Czech Republic.

[4]Department of Applied Chemistry, College of Life Sciences, Ritsumeikan University, Kusatsu 525-8577, Japan.

[5]Graduate School of Pure and Applied Sciences, University of Tsukuba, Tsukuba, Ibaraki 305-8571, Japan.

‡ These authors contributed equally.

Methods

STM experiments: Here, we use a homemade low-temperature scanning tunneling microscope (STM) operating at 4.3 K under ultrahigh vacuum conditions ($P < 5\times10^{-10}$ mbar) and specs LT-AFM operating at 4 K. The Au(111) substrates were cleaned through multiple cycles of sputtering with Ar^+ ions and annealing at 700 K for 15 minutes. The sample temperature was monitored using a combination of a pyrometer and a thermocouple. 5,10,15,20-Tetrakis(2,6-dimethylphenyl) porphyrin Au complex as a Cl^- ion pair[S1] as a precursor was deposited on clean Au(111) surfaces kept at room temperature, followed by annealing to 300 °C. Cl^- was evaporated off before the sublimation. Knudsen cells were used to evaporate precursor molecules. STM images were obtained with chemically etched tungsten tips. Bond-resolved STM (BR-STM) images were conducted using a CO-functionalized tip in constant-height mode (V = 1 mV).

Theoretical calculations:

DFT calculations: The geometry of the gas-phase molecule was optimized in both its neutral and +1 charge states using Density Functional Theory (DFT) as implemented in the FHI-aims code [S2], employing the PBE0 hybrid functional. We examined the neutral and charged species in the gas phase using different XC functionals such as PBE, PBE0, and B3LYP as well as DFT+U ($U_{Au\text{-}d}$=3eV). All these DFT functionals give very similar results. For the neutral molecule, the ground state is a monoradical doublet with a single unpaired electron in a strongly localized d-like orbital, see Figure S11. For the +1 charge state, all applied DFT functionals agree on the closed-shell singlet ground state. This result is not in agreement with the experimental observation of the spin excitation (Figure 1h), which unequivocally indicates an open-shell magnetic ground state. The failure of single-determinant DFT to capture the correct

spin state underscores the strong multireference character of the system, determined by multireference CASCI calculations, see Figure S13.

To account for the influence of the underlying Au(111) surface, the molecule was also optimized on a three-layer Au(111) slab with a rectangular unit cell (8x8), see Figure S8, with the bottom gold layer kept fixed. These slab optimizations were performed using the Perdew–Burke–Ernzerhof (PBE) functional at the Γ-point. To account for dispersion interactions, we employed the Tkatchenko–Scheffler treatment of van der Waals interactions. Furthermore, scalar relativistic effects were included in all calculations through the atomic scalar zeroth-order regular approximation (ZORA) method.

CASCI calculations: Because of the open-shell character of the molecule, we employed the many-body CASCI method for a more accurate description of the wavefunction and energy. The one- and two-body integrals were calculated with the ORCA quantum chemistry package[S3]. Using orbitals obtained from closed-shell DFT with the PBE functional[S4]. From these integrals, the full many-body Hamiltonian was constructed for 12 electrons in 12 orbitals [CAS(12,12)] near the Fermi level. The active space was selected based on natural orbital occupation numbers from preliminary CASCI calculations. Frontier orbitals with occupation numbers deviating significantly from 2 or 0 were included (Figure S7). Different orbital selections (e.g., CAS(8,8), CAS(10,10), CAS(18,12)) were tested, confirming convergence of the singlet–triplet gap (Table S1).

To further account for out-of-CAS dynamical electron correlation, we applied the Quasidegenerate Second-Order N-Electron Valence State Perturbation Theory (QD-NEVPT2)[S5]. For the CASCI calculations, the geometry of the **AuPor** was taken from the structure optimized on the Au(111) surface to account for the effect of mixed valence on the

geometry. In contrast, for **AuPor***, the gas-phase geometry optimized in the +1 charge state was used.

Table 1. Spin-excitation value for the **AuPor*** from the CASCI calculations with different active spaces.

Active Space	$E_T - E_S$ (meV)
CAS(8,8)	7.4
CAS(10,10)	7.2
CAS(12,12)	8.7
CAS(18,12)	8
CAS(8,12)	10

STS simulations: Theoretical d*I*/d*V* maps were computed using the Probe Particle Scanning Probe Microscopy (PP-SPM) code[S6] for a CO-like tip, modeled as a linear combination of PxPy (85%) and s-like (15%) orbitals, without tip relaxation.

Dyson Orbitals: Dyson orbitals were used to interpret the differential conductance (d*I*/d*V*) maps in STM for electron addition and removal processes[S7,S8]. They describe the overlap between the initial and final many-body wavefunctions, thereby capturing the spatial distribution of electronic states involved in tunneling. Dyson orbitals account for the changes in the electronic structure upon electron addition (unoccupied states, positive bias) or removal (occupied states, negative bias), offering a more accurate representation of the local density of states relevant to the STM signal.

Natural Transition Orbitals (NTOs): We calculated NTOs[S9] for the spin-flip process between the ground state and the first excited state, in order to capture the spatial variation of the d*I*/d*V* maps corresponding to IETS spin excitation maps.

Supplementary Figures

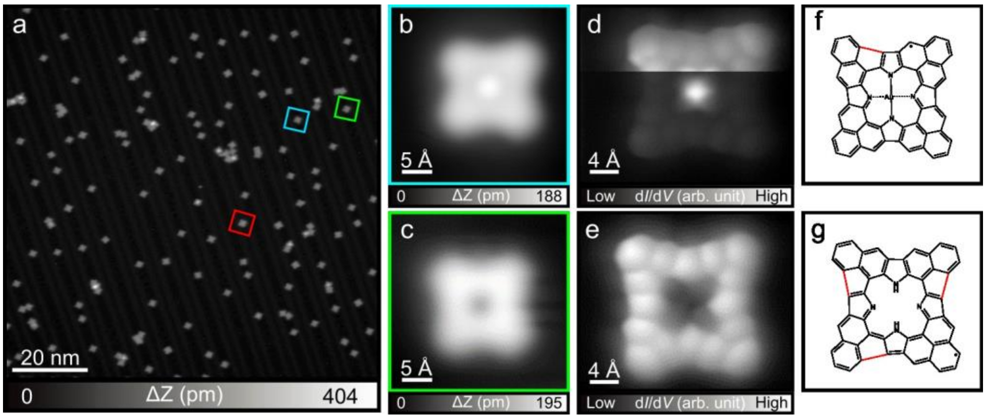

Figure S1. (a) Large-scale STM image after depositing precursor **1** on Au(111) surface followed by annealing to 300 °C. (b, c) Close-up STM images of obtained products in blue and green contours in (a) and (d, e) the corresponding constant-height BR-STM images, respectively. (f, g) The chemical structure of the products in (d) and (e), respectively. Scanning parameter: (a, b, c) V = 0.2 V and I = 10 pA.

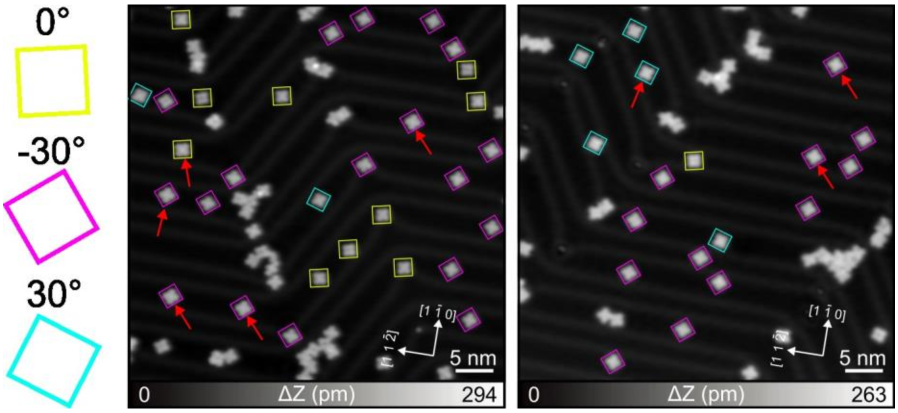

Figure S2. Orientation analysis of Au-porphyrins. Yellow, purple, and light blue square contours indicate different orientations. The yellow contour is defined as 0°, while the purple and light blue contours correspond to –30° and +30° relative to yellow, respectively. Red arrows mark the intact Au-porphyrins. Scanning parameter: V = 0.2 V and I = 10 pA.

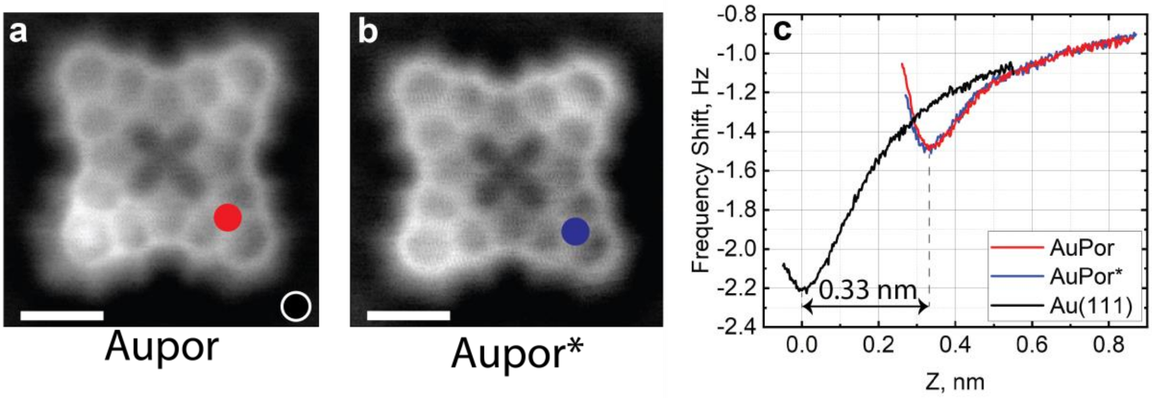

Figure S3. Adsorption heights of molecules. (a,b) CO-tip ncAFM images of **AuPor** and **AuPor***, respectively. Scalebars: 5 Å. (c) ncAFM measurements of $\Delta f(\Delta z)$ for Au(111), **AuPor**, and **AuPor*** at sites shown in panels a and b. The equivalent measurements for **AuPor** and **AuPor*** confirm no difference in their adsorption height above the surface.

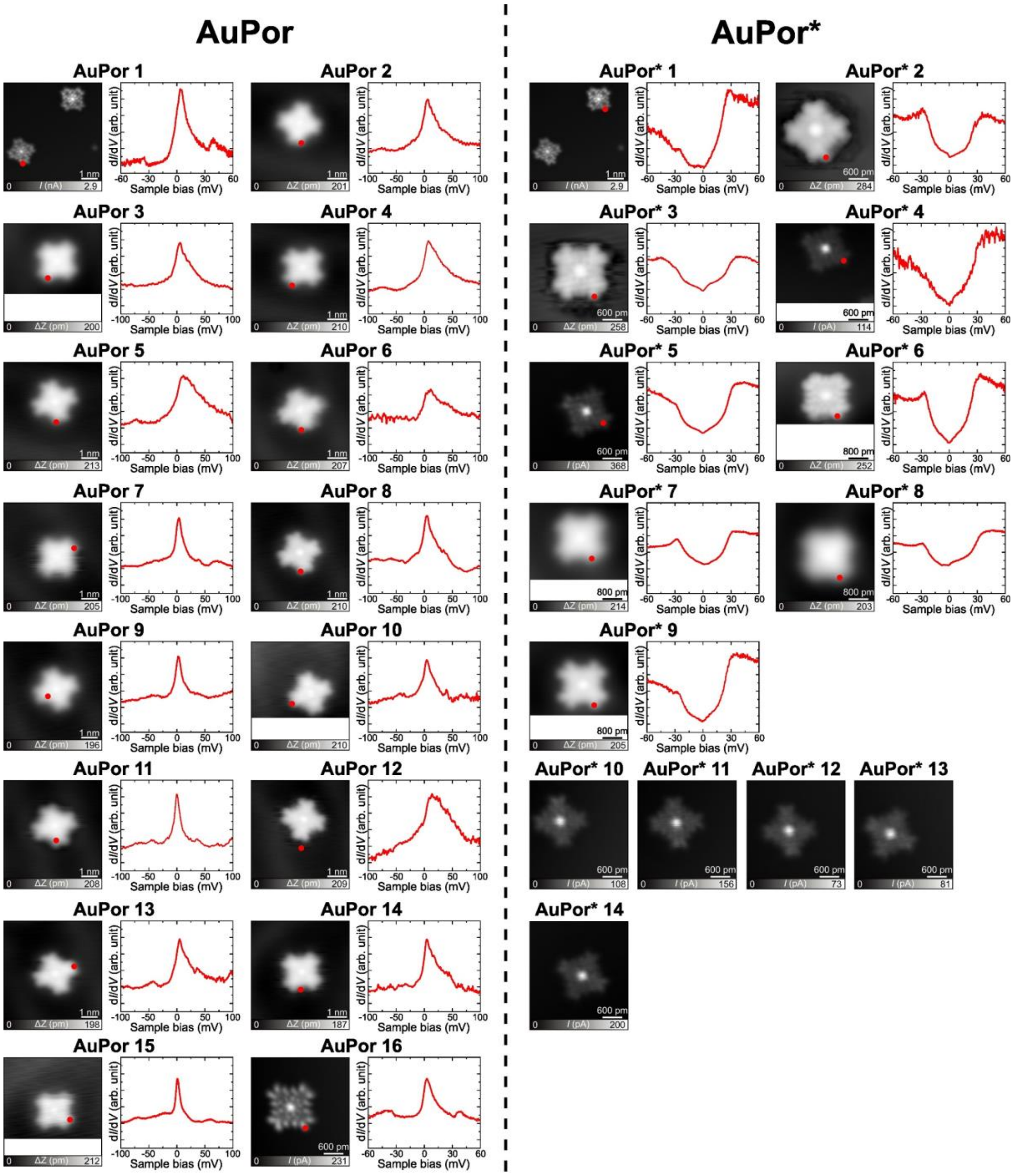

Figure S4. Statistical analysis of all intact Au-porphyrins. From a total of 550 molecules identified from STM survey images across two different sample preparations, we identified around 55 as being intact Au porphyrins with D4 symmetric appearance indicating a yield of approximately 10%. A total of 16 **AuPor** molecules and 14 **AuPor*** molecules were carefully characterized to obtain reliable results. The STM images are shown together with the corresponding d*I*/d*V* spectra acquired at the red-marked sites in the STM images. After examining many examples, we identified an additional method to distinguish **AuPor** and **AuPor***. By comparing **AuPor 16** and **AuPor* 5**, both imaged in constant-height mode at the same bias voltage of 35 mV, the center of **AuPor* 5** appears significantly brighter than its

corners, whereas this contrast is absent in **AuPor 16**. Using this method, we were able to confirm the spin states of **AuPor* 10–14**.

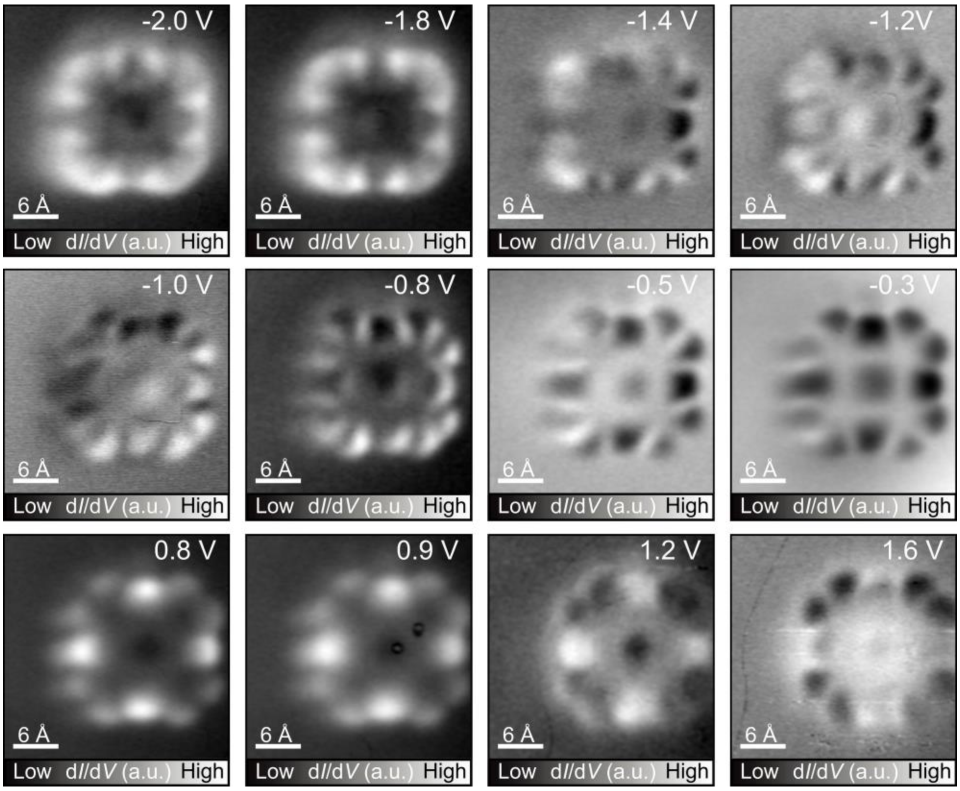

Figure S5. Constant-current dI/dV maps of **AuPor*** taken at different bias with a CO tip.

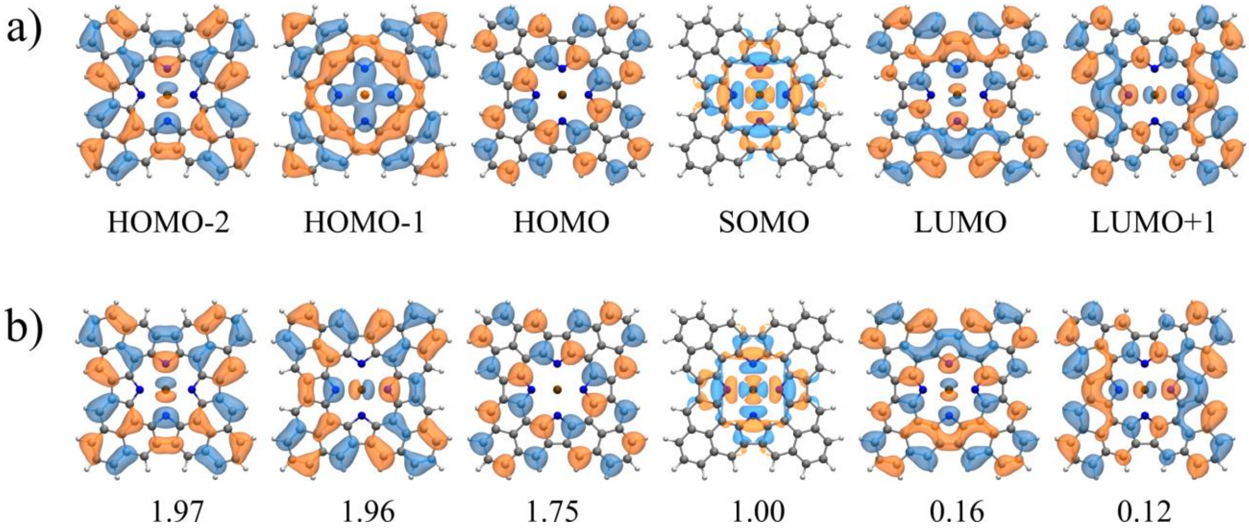

Figure S6. (a) Selected frontier canonical DFT-PBE orbitals used in the active space for CASCI. (b) Multireference natural orbitals obtained from CASCI(12,12) calculations for neutral Au-porphyrin with their occupancies.

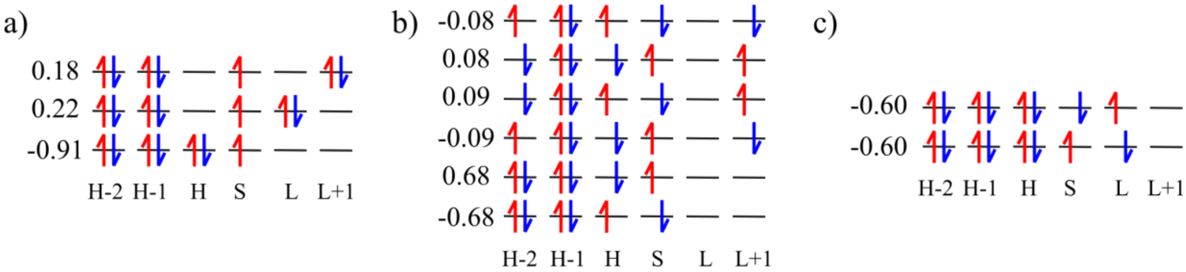

Figure S7. The ground state multireference wavefunctions obtained from CASCI calculations using the optimized geometry obtained from DFT-PBE of the Au-porphyrin in (a) neutral, (b) positively charged, and (c) negatively charged states.

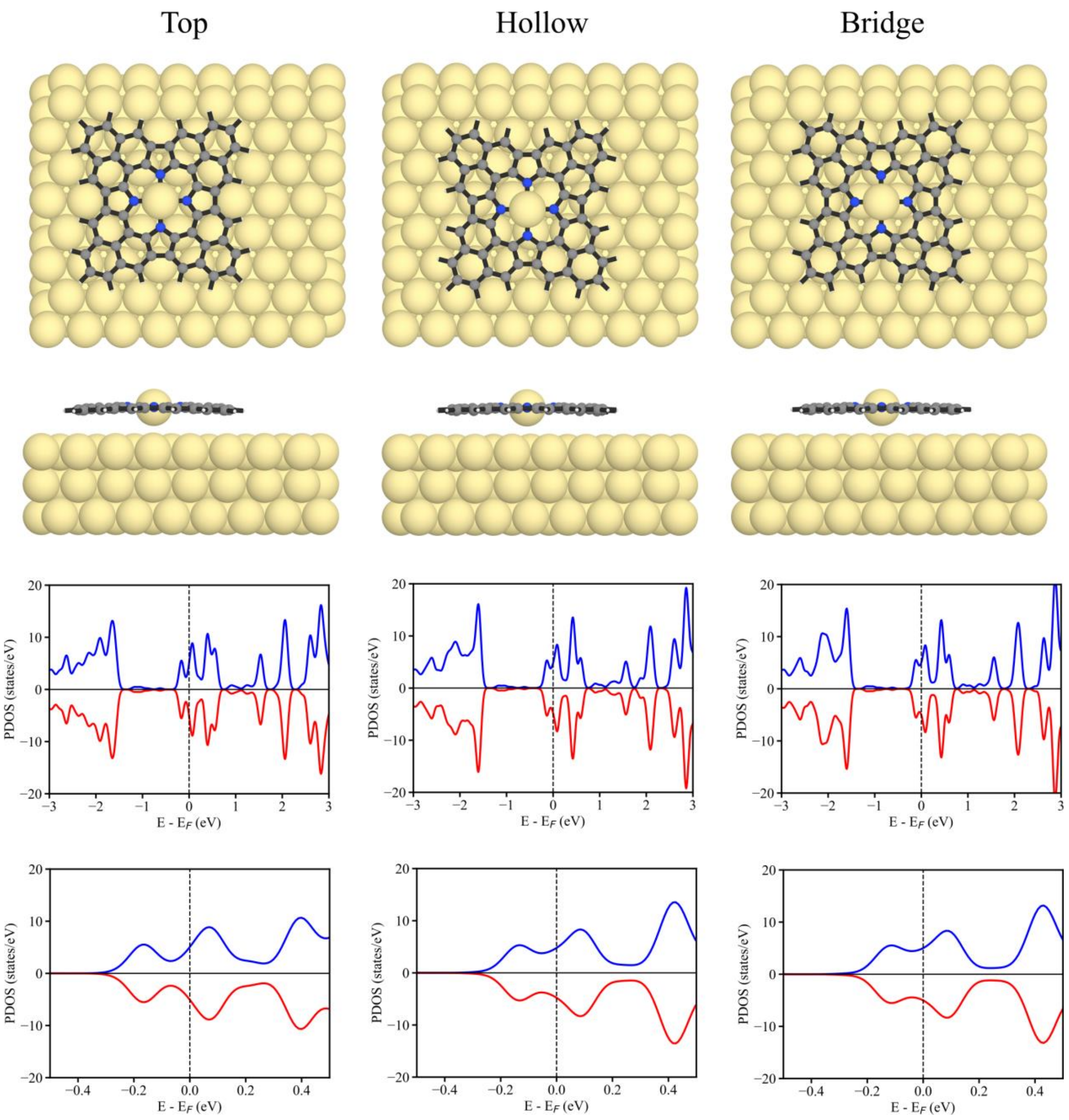

Figure S8. (Top) Top and side views of the optimized structure of Au-porphyrin with a central Au atom positioned at the top, hollow, and bridge adsorption sites on the Au(111) surface obtained from total energy DFT calculations. (Down) Calculated projected density of states (PDOS) of Au-porphyrin for different adsorption sites: top, hollow, and bridge. The results show that PDOS is independent of adsorption configurations. Also, comparison with PDOS of charged +1 molecule in gas-phase shown in Figure S9 demonstrates the absence of hybridization of molecular orbitals with electronic states of the substrate.

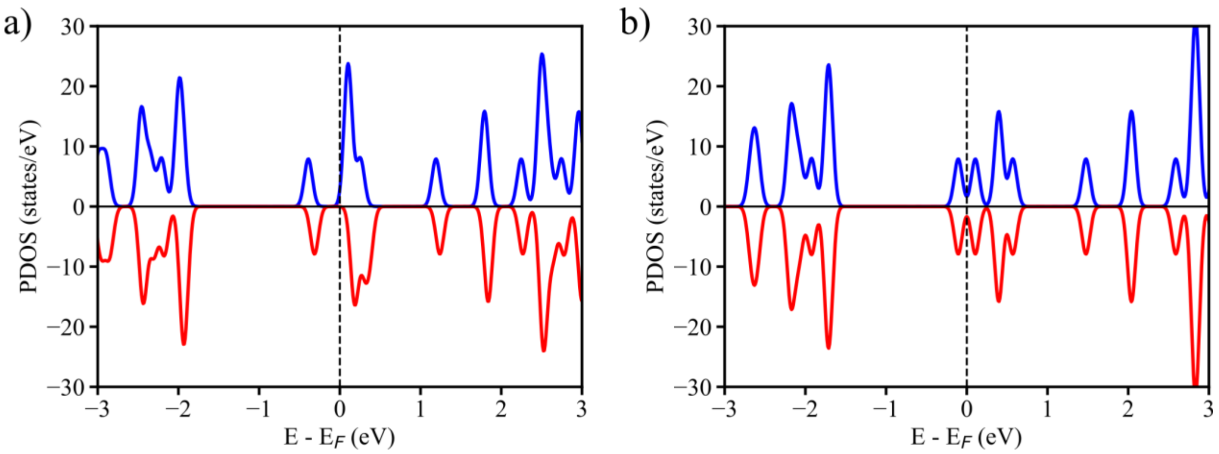

Figure S9. Projected density of states (PDOS) of Au-porphyrin in the gap phase for (a) the neutral state and (b) the +1 charged state obtained from DFT-PBE calculation.

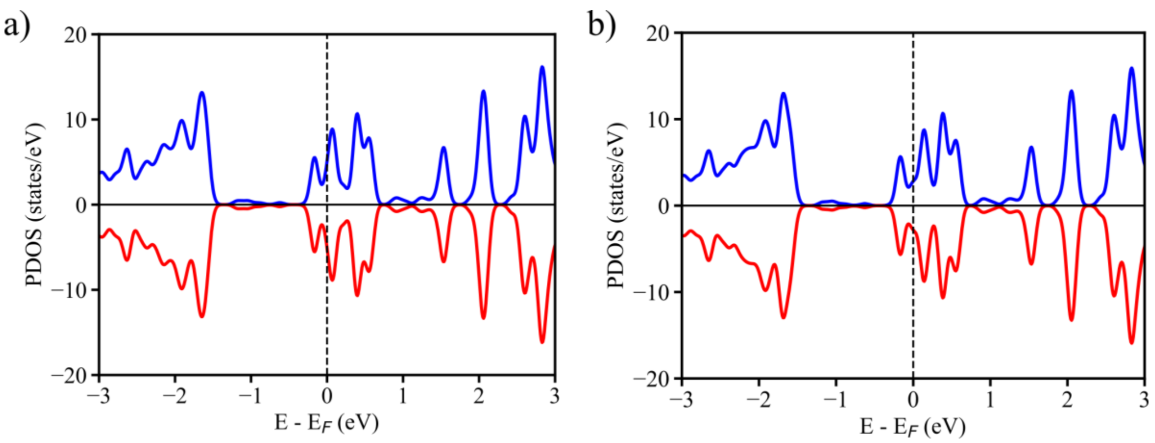

Figure S10. Projected density of states of Au-porphyrin at the top adsorption site, calculated using (a) PBE and (b) PBE+U with U= 3 eV.

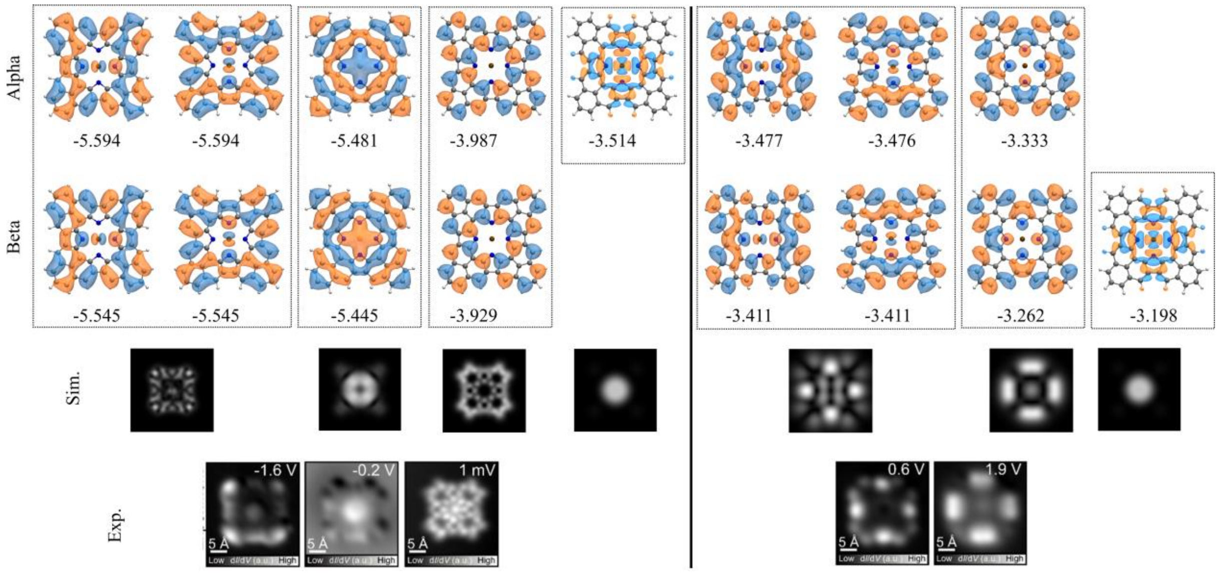

Figure S11. Canonical DFT–PBE molecular orbitals of **AuPor** with the corresponding simulated d*I*/d*V* and experimental d*I*/d*V* maps.

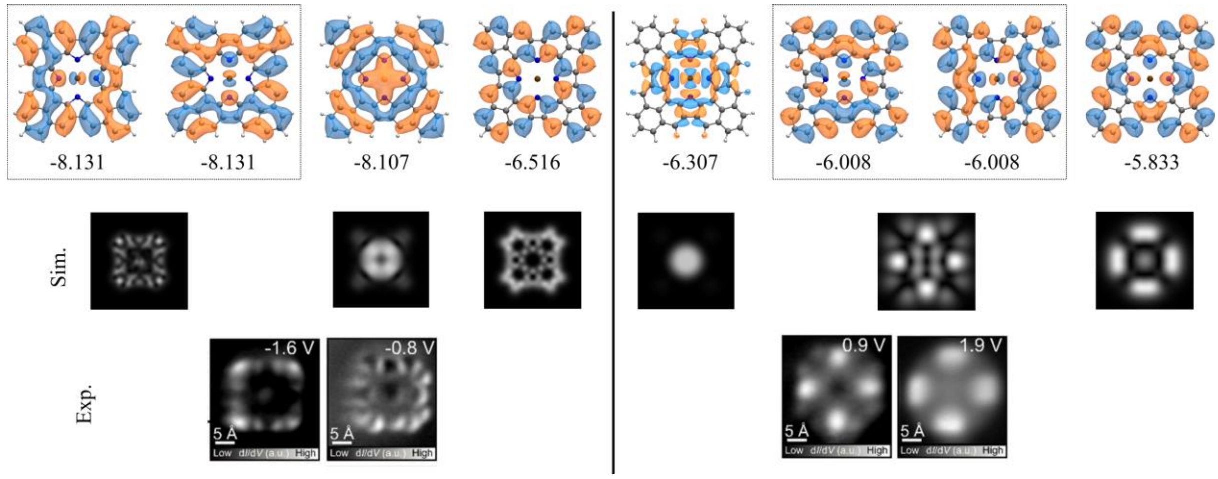

Figure S12. Canonical DFT–PBE molecular orbitals of **AuPor*** with the corresponding simulated d*I*/d*V* and experimental d*I*/d*V* maps.

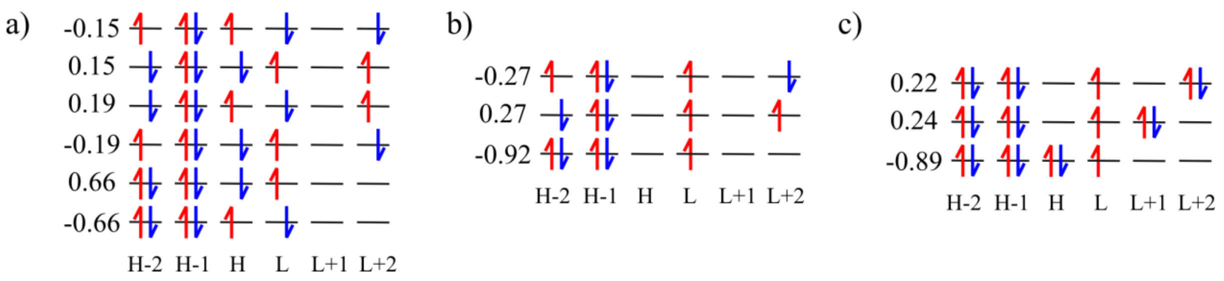

Figure S13. Multireference wavefunctions obtained from CASCI(12,12) calculations for the ground state of **AuPor***, considering fully optimized geometry from DFT-PBE with one electron less in the ground state with respect to the reference **AuPor** due to the electron transfer to Au(111) substrate, in (a) neutral, (b) positively charged, and (c) negatively charged states.

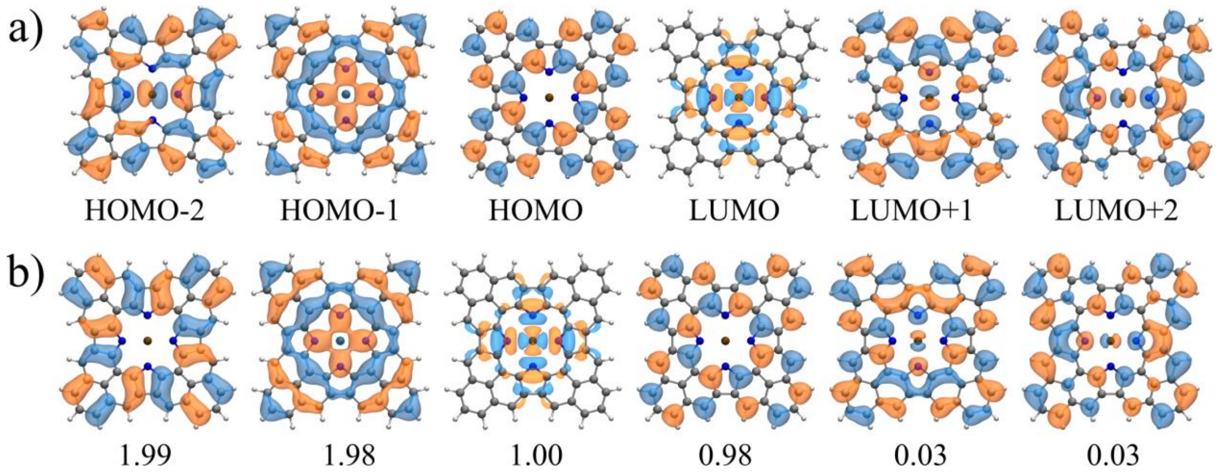

Figure S14. (a) Selected canonical frontier DFT-PBE orbitals used as basis for CASCI(12,12). (b) Multireference natural orbitals obtained from CASCI(12,12) calculations for **AuPor*** with their occupancy revealing the presence of two unpaired electrons.

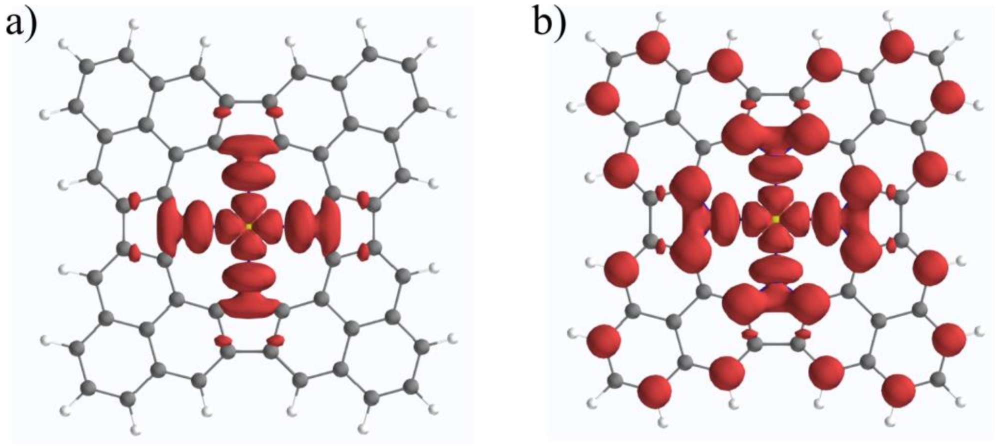

Figure S15. Calculated unpaired electron density obtained from multireference CASCI calculations for (a) **AuPor** and (b) **AuPor***, isovalue 0.001 e/Å³.

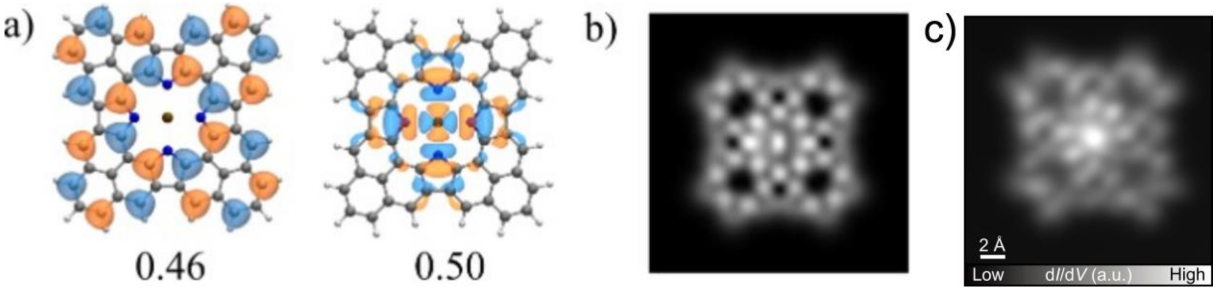

Figure S16. (a) Natural transition orbitals (NTOs) corresponding to transition from the singlet ground state to the first excited triplet state; (b) Simulated d*I*/d*V* maps of NTOs with CO-like tip for **AuPor***; (c) experimental spin excitation map of **AuPor***.

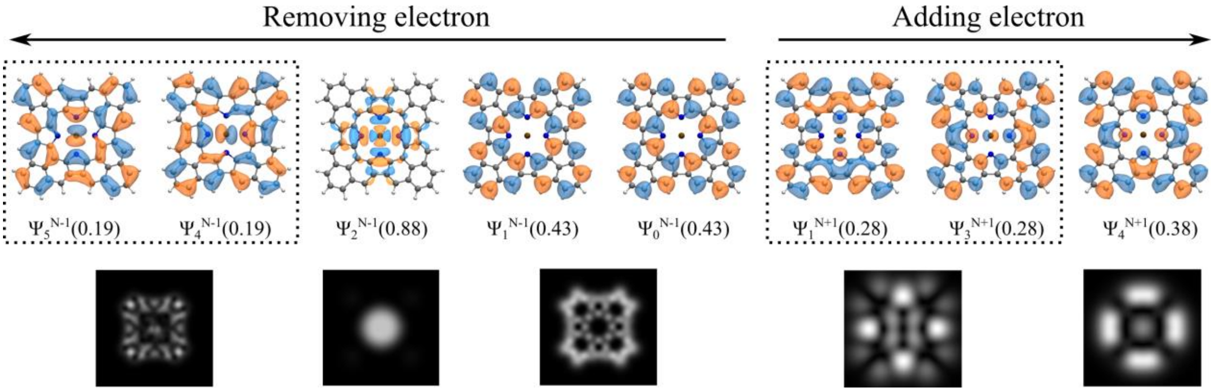

Figure S17. Dyson orbitals for electron removal (left) and electron addition (right) with the norm of the overlap given in parentheses for **AuPor**.

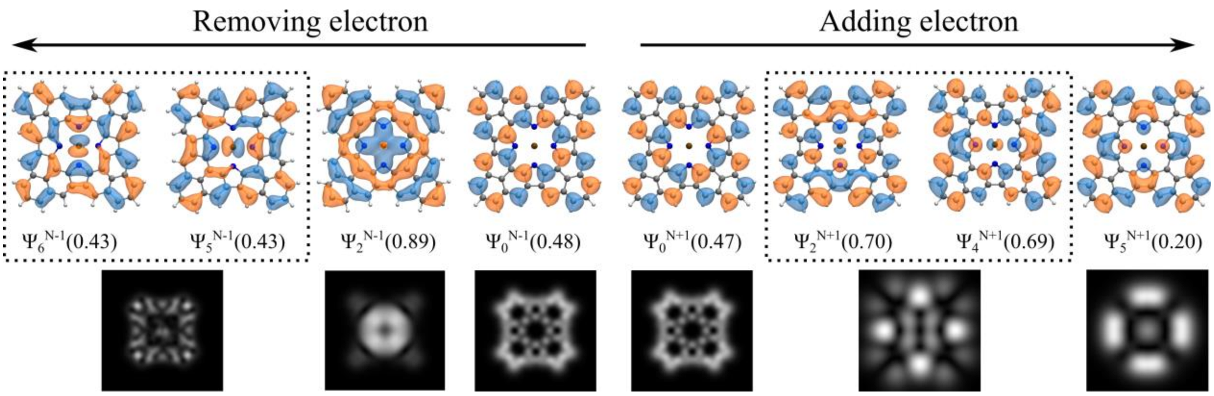

Figure S18. Dyson orbitals for electron removal (left) and electron addition (right) with the norm of the overlap given in parentheses for **AuPor***.

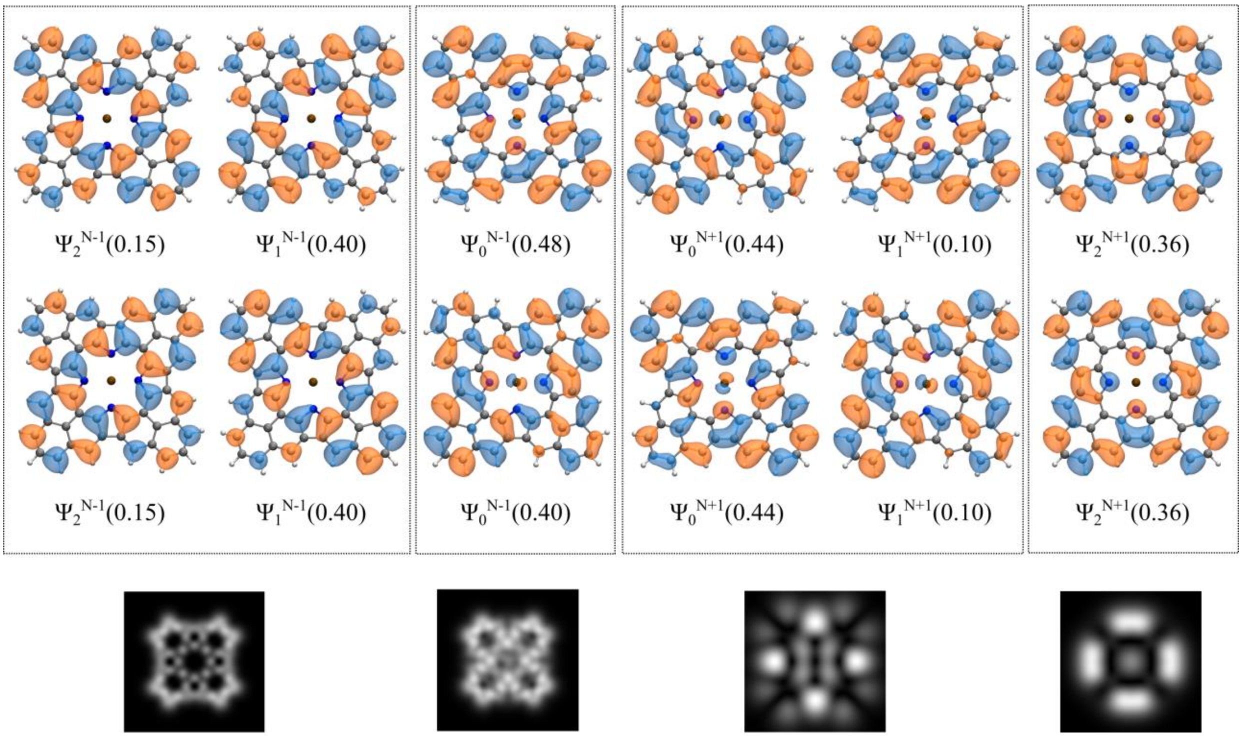

Figure S19. Dyson orbitals for electron removal and electron addition with the norm of the overlap given in parentheses for negatively -1e charged Au-Porphyrin from doubly degenerated neutral ground states.

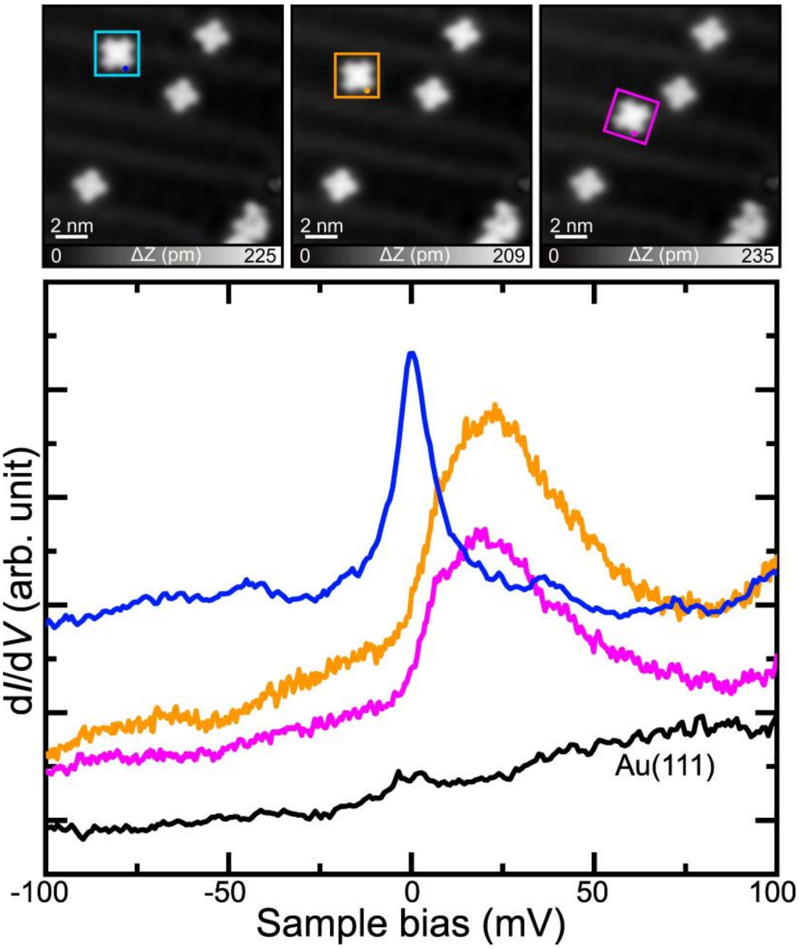

Figure S20. The manipulation of **AuPor** and the corresponding d*I*/d*V* spectra. After manipulation, the corresponding d*I*/d*V* spectrum shows that the peak shifts to higher energy and becomes broadened. Scanning parameter: $V = 0.2$ V and $I = 10$ pA.

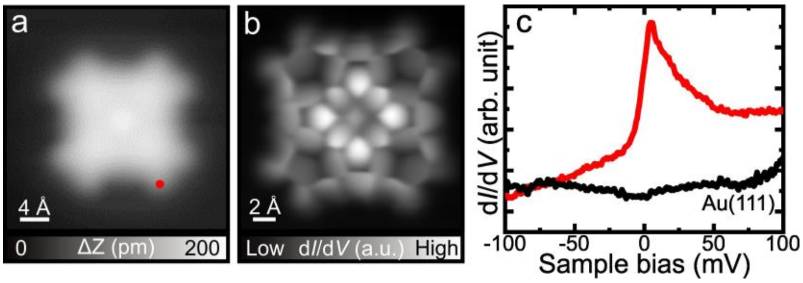

Figure S21. (a) Close-up STM image of **AuPor**. (b) The corresponding constant-height BR-STM image. (c) d*I*/d*V* spectrum taken over the marked site in (a). Scanning parameter: (a) $V =$ 0.2 V and $I = 10$ pA. (b) $V = 1$ mV and $V_{\mathrm{mod}} = 10$ mV.

II: Singly impurity Anderson model (SIAM)

The Single Impurity Anderson Model (SIAM) is employed to study the **mixed-valence regime** and the suppression of the net spin for a molecule with a single unpaired electron in a single site coupled to a metal surface. The model considers a single molecular orbital coupled to an effective chain that models the metallic substrate. The SIAM describes a single magnetic orbital (impurity) here corresponding to the unpaired electron in the Au-porphyrin molecule coupled to a metallic substrate (Au(111)) to understand What happens to the molecular spin when the orbital energy lies very close to the Fermi energy of the metal?

The total Hamiltonian $\hat{H}$ is expressed as the sum of three terms:

$$\hat{H} = \hat{H}_{\text{imp}} + \hat{H}_{\text{chain}} + \hat{H}_{\text{int}} ,$$

where $\hat{H}_{\text{imp}}$ describes the impurity, $\hat{H}_{\text{chain}}$ the metallic chain, and $\hat{H}_{\text{int}}$ the hybridization between them.

The impurity corresponds to the singly site model,

$$\hat{H}_{\text{imp}} = \Delta \sum_{\sigma} \hat{d}_{\sigma}^{\dagger} \hat{d}_{\sigma} + U \hat{d}_{\uparrow}^{\dagger} \hat{d}_{\downarrow}^{\dagger} \hat{d}_{\downarrow} \hat{d}_{\uparrow} ,$$

where U is Coulombic repulsion in the SOMO orbital ($U \approx 2.0\,\text{eV}$ in this work), Δ is Energy level of the impurity, and $\hat{d}_{\sigma}^{\dagger}$, $\hat{d}_{\sigma}$ are Creation and annihilation operators for the impurity level with spin σ. Impurity Site (Molecule) is modeled as a single orbital that can host 0, 1, or 2 electrons. The energy of this orbital is denoted by Δ. If two electrons occupy the orbital, they experience a Coulomb repulsion U, analogous to the on-site Hubbard interaction in transition-metal complexes.

The substrate is modeled as a finite one-dimensional finite chain (here 5 sites) with delocalized electrons. The chain mimics the continuous density of states near the Fermi level of a metal and described by a tight-binding Hamiltonian:

$$\hat{H}_{\text{chain}} = -T \sum_{m=1}^{N-1} \sum_{\sigma} \hat{c}^{\dagger}_{m+1,\sigma}\, \hat{c}_{m,\sigma} + \epsilon \sum_{m=1}^{N} \sum_{\sigma} \hat{c}^{\dagger}_{m,\sigma}\, \hat{c}_{m,\sigma}\,,$$

where T is the hopping parameter between adjacent sites ($T = 0.2$ eV for metallic behavior), ϵ is the On-site energy for each site, which characterizes the Fermi energy of the chain. For simplicity, $\epsilon = 0$, and $\hat{c}^{\dagger}_{m,\sigma}$, $\hat{c}_{m,\sigma}$ are Creation and annihilation operators for electrons at site m with spin σ. The hopping between neighboring metal sites controls the bandwidth and thus the metallic character.

The molecule interacts with the surface through a hopping term which allows electrons to tunnel between the molecular orbital and the nearest atom of the metal chain. The hybridization between the impurity and the metallic chain is described by:

$$\hat{H}_{\text{int}} = -t \sum_{\sigma} \hat{c}^{\dagger}_{1,\sigma}\, \hat{d}_{\sigma} + h.c.,$$

where t is the hopping parameter coupling the impurity to the first site of the chain. This term physically represents the overlap between molecular and substrate wavefunctions.

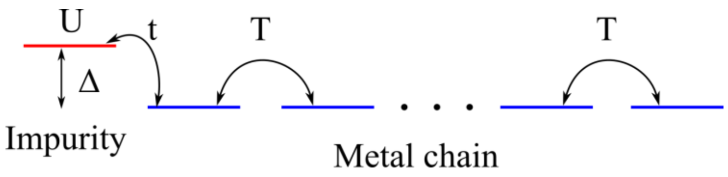

Figure S22. Scheme of single impurity Anderson model depicting the coupling of single impurity level with the linear chain which mimic the density of states of the metal.

Basis Set and Many-Body Representation: The SIAM Hamiltonian is expressed in second quantization, which naturally captures many-body electron correlations. We work in the full many-body Fock space, where basis states are defined by the occupancy of each spin-orbital in the system (impurity + chain).

Since our model contains 1 impurity orbital + 5 chain sites = 6 spatial orbitals, each with spin up/down, the total number of spin-orbitals is 12. The Fock space dimension grows combinatorially with electron number. We consider all possible electron distributions with total electron count corresponding to half-filling (6 electrons for 6 sites). The Hamiltonian is then constructed and diagonalized exactly within this full configuration interaction (CI) basis, ensuring that all many-body effects, including charge fluctuations, and correlation are explicitly included.

We carried out a parametric study of the impurity model to explore different regimes by solving the total Hamiltonian through exact diagonalization. For simplicity, the inter-site hopping was fixed at $T = 0.2$ eV, a value sufficient to reproduce metallic behavior in the finite chain. Figure S23 presents the variation of the impurity-level occupancy (N) and net spin (S^2) for different alignments of the impurity level relative to the Fermi level of the metallic chain. The parameter Δ was varied from –100 meV to +100 meV, effectively simulating the shifting of the impurity level across the Fermi level. Within this range, we observe how the coupling t controls both the population of the impurity and its spin value, revealing their close correlation. At $t = 0$, as expected, the impurity hosts a single electron with $S^2 = 0.75$, corresponding to a pure doublet radical state. When the interaction t is introduced, the impurity occupancy begins to fluctuate. For Δ values below the Fermi energy, hybridization with the chain increases as the impurity level approaches the Fermi level ($\Delta \rightarrow 0$), and changes in t become increasingly pronounced near this limit. Once the impurity level is pinned close to the Fermi level, charge fluctuations in and out of the chain stabilize a half-filled impurity, giving rise to the mixed-valence regime. As Δ shifts towards positive values, the impurity eventually discharges, yielding $N = 0$ and $S^2 = 0$, thus restoring spin as a good quantum number despite sizable coupling t. Since the experimentally observed impurity state appears near the Fermi level, we further computed the density of states (DOS) for the combined chain–impurity system. Figure S24a–c illustrate the

DOS for small negative Δ values approaching the Fermi level with a weak hybridization t = 0.01 eV. Even in this weak-coupling regime, the system enters the mixed-valence regime, characterized by charge transfer (N < 1) and a reduction of the radical character ($S^2 < 0.75$). Figure S24d–f then show the effect of increasing t at fixed Δ < 0, highlighting stronger hybridization between the impurity and the metallic chain. Under these conditions, the impurity state broadens, charge transfer is enhanced, and the radical character is fully quenched, closely resembling the situation encountered in experiments. This analysis confirms that the hybridization and pinning of the molecular orbital near the Fermi level lead to a mixed-valence regime, effectively quenching the local magnetic moment of **AuPor** and explaining the absence of Kondo physics and interaction with the $NiCp_2$ tip. The SIAM results are consistent with the DFT-PDOS (Figure S8), which shows the SOMO pinned at E_F. This alignment, combined with finite hybridization (t ≈ 0.01–0.1 eV), drives the system into a mixed-valence regime where charge fluctuations quench the local spin, as observed experimentally for **AuPor**.

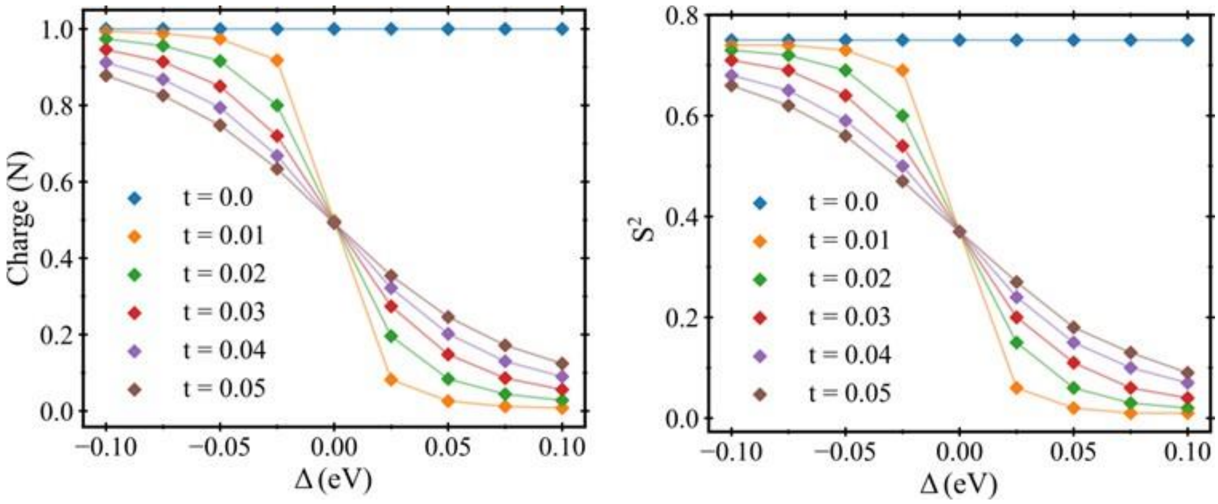

Figure S23. Variation of impurity-level occupancy and net spin. Panels show (a) the electron number and (b) the spin value of the molecular level as functions of Δ, for different coupling strengths t with fixed U = 2.0 eV.

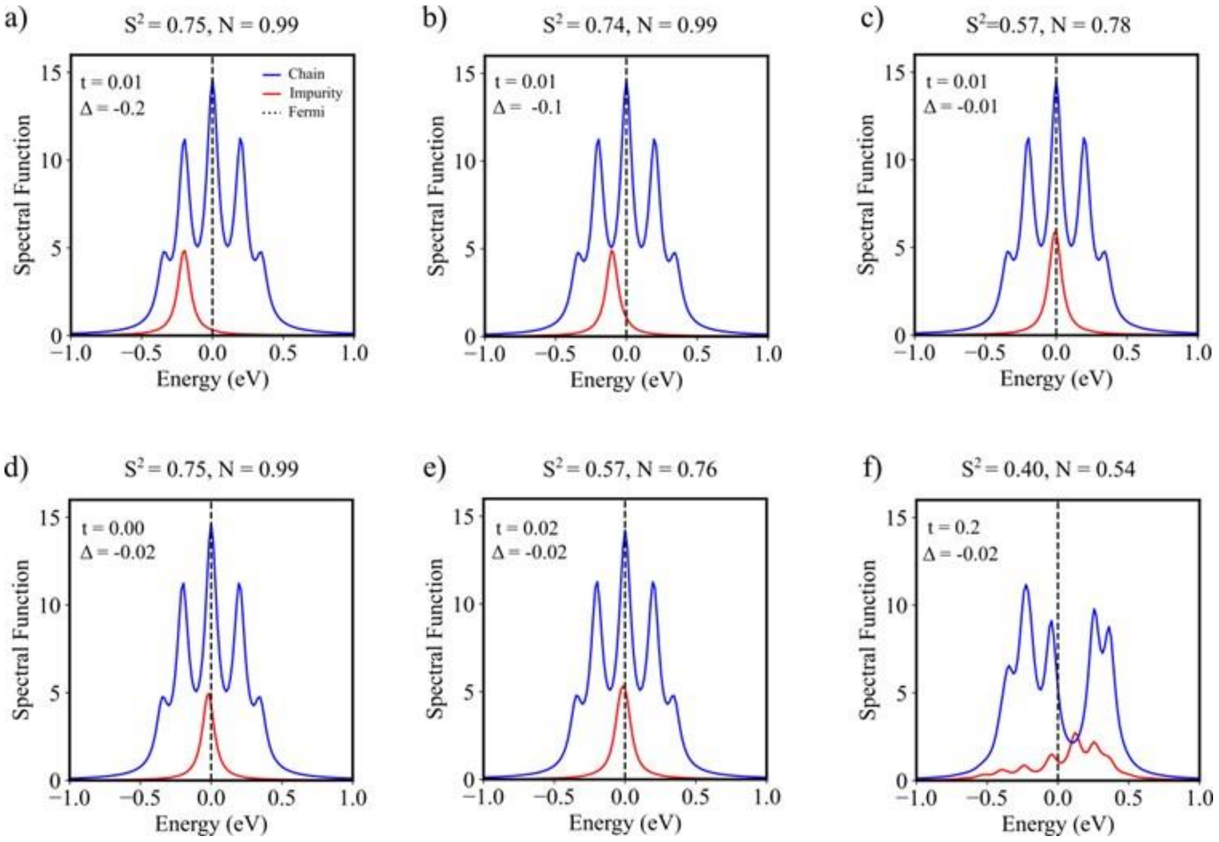

Figure S24. Calculated density of states (DOS) of the impurity model. (a–c) DOS for different impurity-level alignments Δ relative to the Fermi level (dashed line) at fixed coupling t = 0.01 eV, along with occupancy (N) and spin expectation value (S^2). Approaching the Fermi level drives the system into a mixed-valence regime with charge transfer (N < 1) and loss of radical character ($S^2 \ll 0.75$). (d–f) DOS, N, and S^2 as functions of increasing coupling t at fixed Δ = –0.02 eV, showing state broadening, charge transfer, and quenching of the radical character in the impurity state.

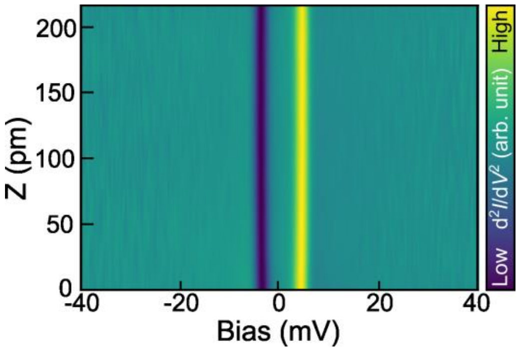

Figure S25. Height-dependent map composed of a series d^2I/dV^2 spectra acquired with a $NiCp_2$ tip on a bare Au(111) surface.

III: Spin model for the interaction between the $NiCp_2$ tip and molecule

To interpret the variation observed in the experimental inelastic electron tunneling spectroscopy (IETS) signal using the $NiCp_2$-functionalized tip, we combine the Heisenberg spin model, which describes spin-spin interactions, with cotunneling theory. This approach enables us to simulate the corresponding inelastic tunneling current[S10,S11]. The Heisenberg model accounts for the exchange interaction $J_{\mathrm{Nc},i}(z)$ between the spin $\hat{S}_i$ of individual spin-½ sites within the molecule, and the spin $\hat{S}_{Nc} = 1$ associated with the $NiCp_2$ tip. This exchange interaction depends on the vertical tip-sample distance z.

The spin Hamiltonian of the $NiCp_2$ tip is described by a uniaxial out-of-plane magnetic anisotropy, $\hat{H}_{\mathrm{Nc}} = D\hat{S}_{Nc,z}^2$, where the anisotropy constant is D = 4 mV. The molecular spin system is modeled as a set of coupled spin-½ centers with a Hamiltonian $\hat{H}_M = \sum_{i,j} J_{i,j}\, \hat{S}_i \cdot \hat{S}_j$, where the exchange coupling parameters $J_{i,j}$ are fitted to reproduce the molecular spin excitation obtained from experiment. The exchange interaction between the $NiCp_2$ tip spin and each molecular spin is given by $\hat{H}_{\mathrm{int}}(z) = \sum_i J_{\mathrm{Nc},i}\,(z)\hat{S}_{Nc} \cdot \hat{S}_i$. The exchange interaction between the $NiCp_2$ tip spin and the molecular spin sites was modeled as a distance-dependent Heisenberg coupling as $J_{\mathrm{Nc},i}(z) = J_{0,i}\exp(-\lambda z)$, where $J_{0,i}$ is the exchange coupling amplitude at the closest tip–sample distance and λ is the decay constant. In the simulations, $J_{0,i}$ was varied in the range 0–8 meV to reproduce the experimentally observed evolution of the IETS spectra as a function of tip height. The decay parameter λ was not treated as a free parameter but was constrained by the spatial decay of the electronic hybridization between the $NiCp_2$ tip orbital and the molecular spin-carrying orbitals. Specifically, λ was chosen such that $J_{\mathrm{Nc},i}(z)$follows the same exponential decay as the square of the tunneling matrix element (hopping amplitude), which was independently obtained from the overlap between the tip and molecular electronic states. This procedure is justified by perturbative exchange theory, where the magnetic exchange interaction scales as the second order of the hopping amplitude (chain-

rule dependence), and is consistent with established models of tip–molecule exchange coupling in STM-based IETS measurements on metal surfaces [S10,S11]. Performing a fully ab initio evaluation of the tip–molecule exchange interaction would require a detailed treatment of the extended tip–substrate–molecule junction at multiple tip heights, which is currently computationally prohibitive and this treatment was not required, as the exchange interaction is perturbative in the tunneling regime and is reliably captured by the constrained exponential model employed here. Combining all contributions, the total spin Hamiltonian of the system becomes:

$$\hat{H}_{M,\mathrm{Nc}}(z) = \hat{H}_M + \hat{H}_{Nc} + \hat{H}_{int}(z) = \sum_{i,j} J_{i,j}\,\hat{S}_i \cdot \hat{S}_j + D\hat{S}_{Nc,z}^2 + \sum_i J_{\mathrm{Nc},i}\,(z)\hat{S}_{Nc} \cdot \hat{S}_i \ . \qquad Eq(1)$$

However, the eigenvalues of the Hamiltonian in Eq. (1) cannot be directly correlated with the experimentally observed IETS peaks, as the tunneling intensities associated with specific spin excitations can vary significantly. To quantitatively capture the relative intensities of these features, we employ cotunneling theory to compute the inelastic tunneling current through the spin system described by Eq. (1), accounting for its coupling to tip and surface.

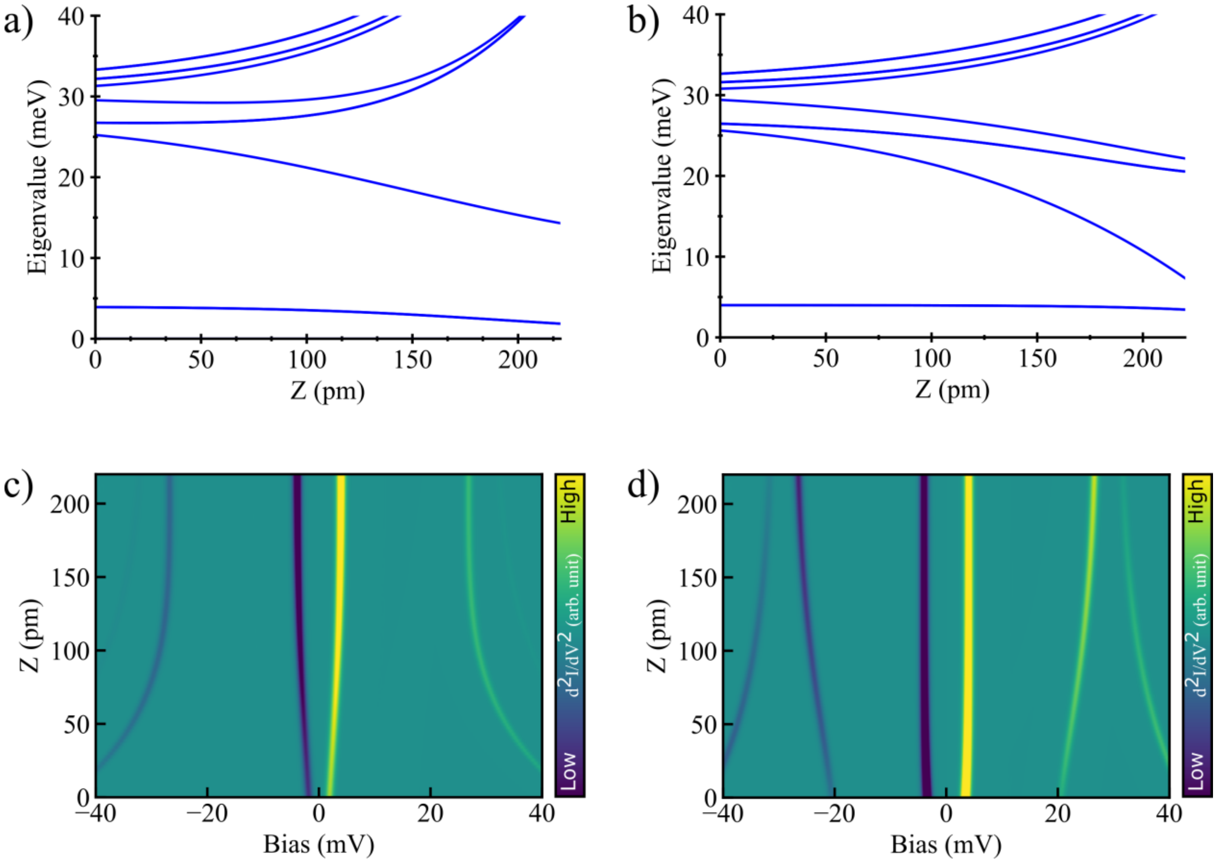

Figure S26. (a,b) Eigenvalues of the Heisenberg Hamiltonian in Eq. 1 for the interaction with a $NiCp_2$ tip as a function of coupling strength *J(z)*, shown for (a) the localized central Au spin and (b) the highly delocalized molecular spin on the molecular periphery. (c,d) Simulated d^2I/dV^2 spectra for the interaction with a $NiCp_2$ tip as a function of coupling strength *J(z)*, shown for (c) the localized central Au spin and (d) the highly delocalized molecular spin on the molecular periphery.

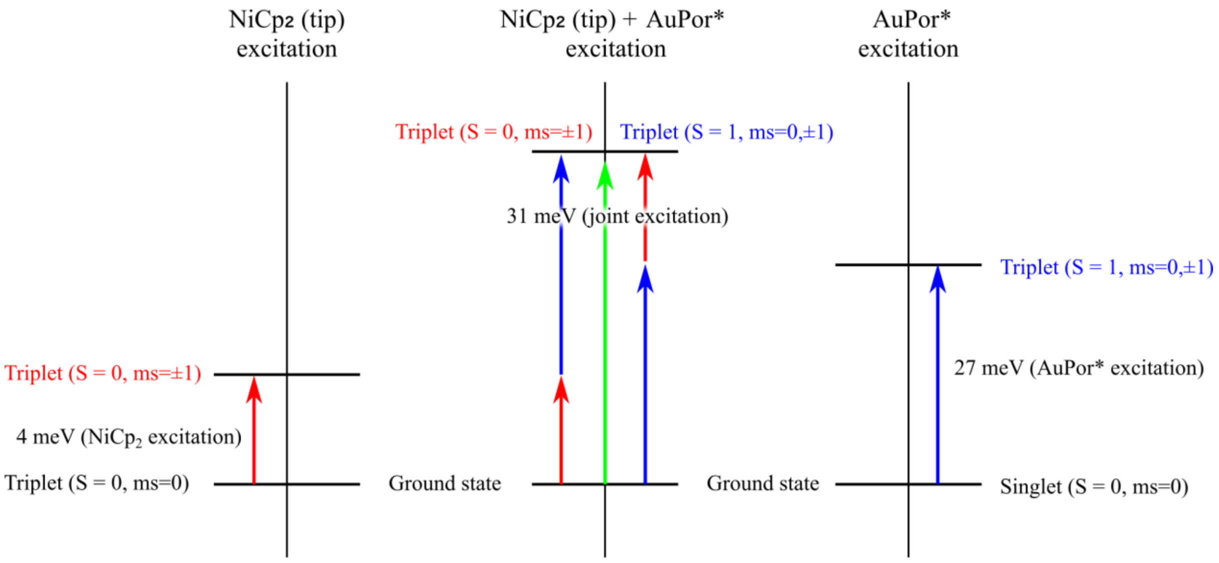

IETS Spin Excitations with NiCp2-Functionalized Tip

Figure S27. Energy diagram of spin excitations probed by IETS with a $NiCp_2$-functionalized tip. Schematic representation of the electronic transitions observed in d^2I/dV^2 spectra. The diagram illustrates the independent spin-1 excitation of the $NiCp_2$ tip (4 meV), the singlet-triplet magnetic excitation of the **AuPor*** molecule (27 meV), and the joint excitation (31 meV) resulting from the simultaneous transition of both the tip and molecular spins.

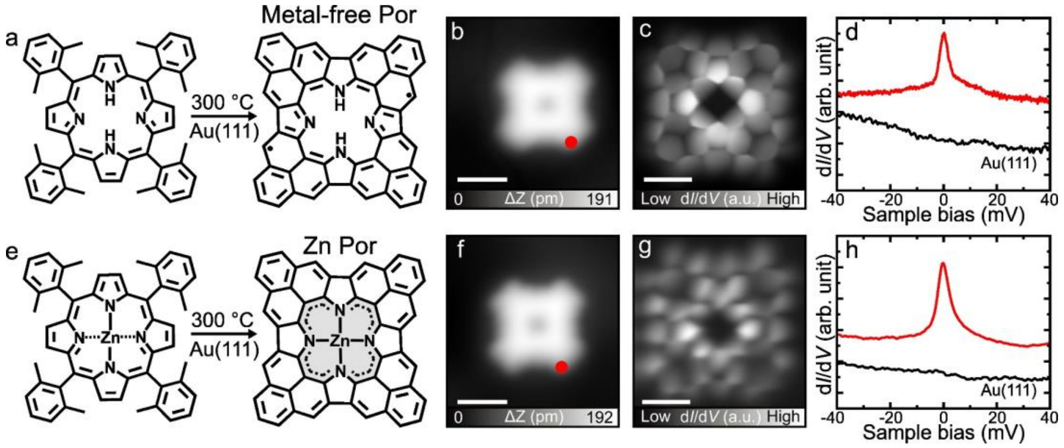

Figure S28. Characterization of metal-free and Zn porphyrins. (a) Synthetic route of phenalenyl expanded metal-free porphyrin. (b,c) STM topography and the corresponding BR-STM image of metal-free porphyrin, respectively. (d) dI/dV spectrum taken over the marked site in (b). (e) Synthetic route of phenalenyl expanded Zn porphyrin. (f,g) STM topography and the corresponding BR-STM image of Zn porphyrin, respectively. (h) dI/dV spectrum taken over the marked site in (f). Scanning parameters: (b,f) V = 0.2 V, I = 10 pA. (c,g) V = 1 mV, V_{mod} = 10 mV. Scale bars: (b,f) 1 nm. (c,g) 5 Å.

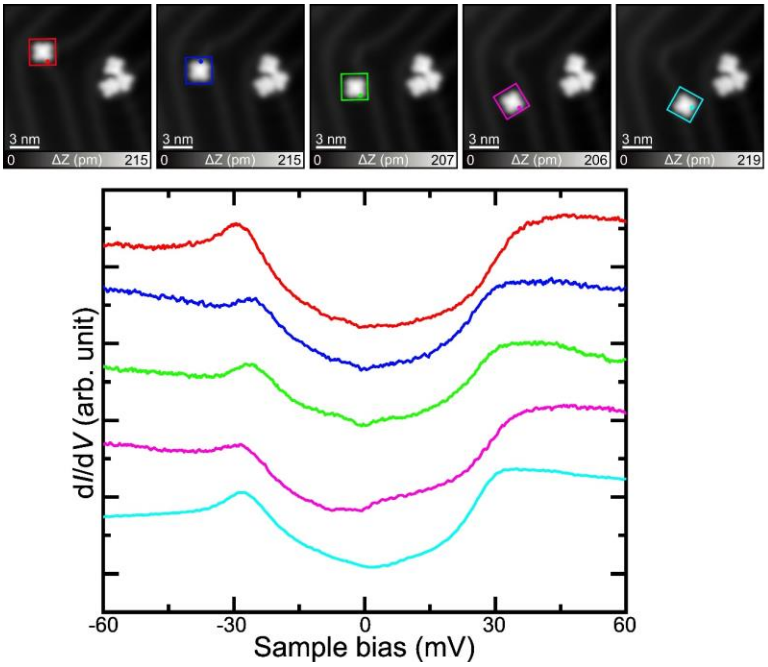

Figure S29. The manipulation of **AuPor*** and the corresponding dI/dV spectra. After manipulation, the corresponding dI/dV spectra show almost no change, indicating that the manipulation cannot alter the spin state. Scanning parameter: V = 0.2 V and I = 10 pA.

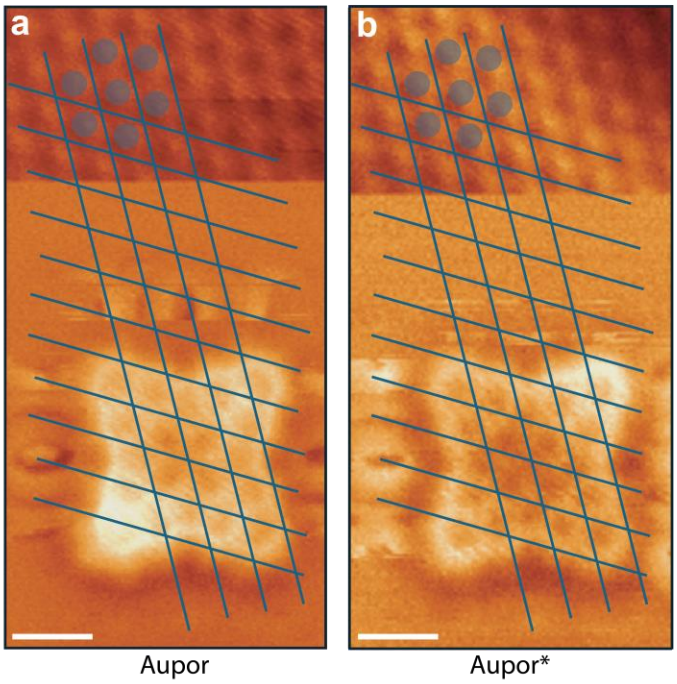

Figure S30. Adsorption positions on the Au(111) surface. a,b) CO-tip ncAFM images taken at different heights revealing the atomically resolved Au(111) surface (upper) and **AuPor** or **AuPor*** chemical structure (lower), respectively. From these hybrid images, we can determine the adsorption position of the molecules. There is no detectable difference between **AuPor** and **AuPor***. Scalebars: 5 Å.

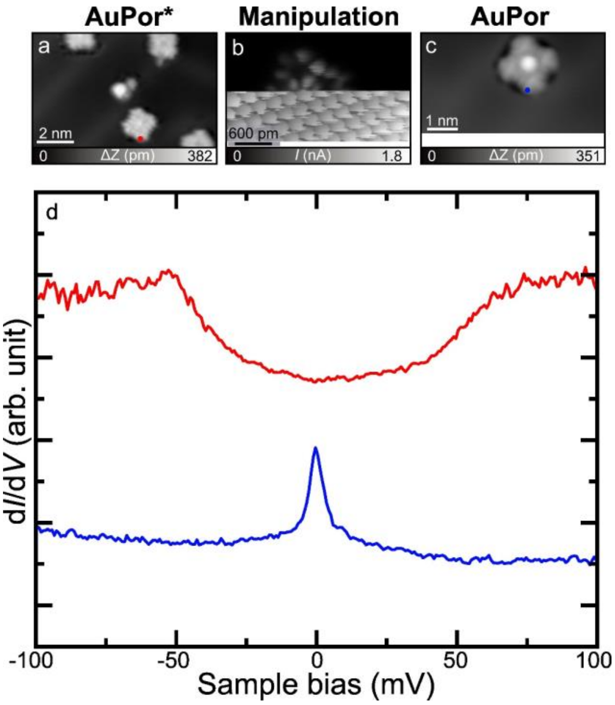

Figure S31. Uncontrolled conversion between the two species. (a, b, c) STM images of Au-porphyrin before manipulation, during manipulation, and after manipulation. (d) d*I*/d*V* spectra acquired at the red and blue dots marked in (a) and (c), respectively. First, we confirmed that the Au-porphyrin molecule in (a) corresponds to **AuPor***: the red d*I*/d*V* spectrum in (d) exhibits a dip feature. We then reduced the tip–sample distance and scanned this **AuPor*** species. When the tip passed over the central site of **AuPor***, the molecule was suddenly picked up by the tip, likely due to a strong interaction between the Au center and the tip apex. After retracting the tip, the molecule dropped back onto the surface and converted into **AuPor** (c), as evidenced by the emergence of a resonance peak near the Fermi level in the d*I*/d*V* spectrum (blue curve in d).

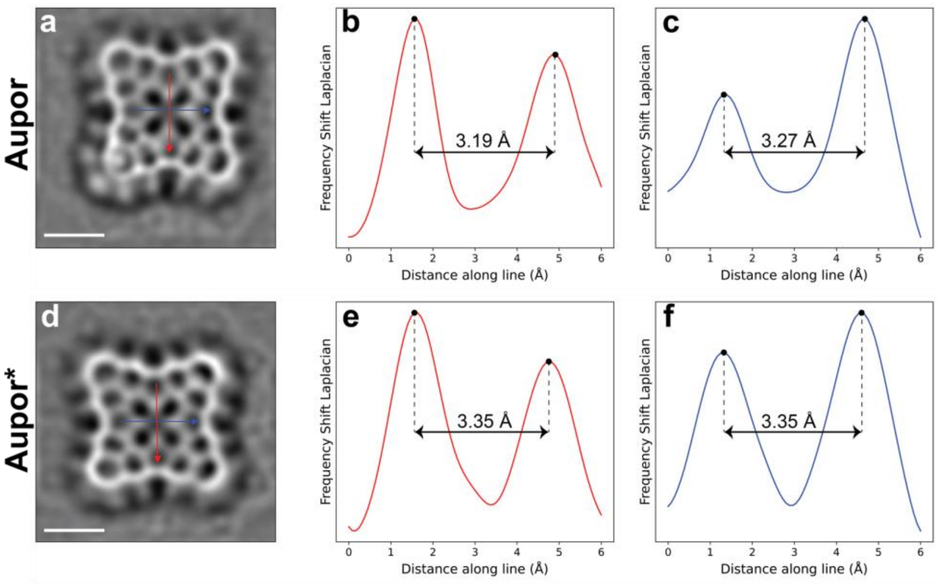

Figure S32. NcAFM comparison of distances at the core of **AuPor** and **AuPor***. (a) Laplace-filtered ncAFM image of **AuPor** acquired with a CO tip. (b, c) Line profiles along the paths shown by red and blue arrows, respectively, in (a) The distance between local AFM maxima was used to provide an indication of bond lengths at the molecule core. (d) Laplace-filtered image of **AuPor*** acquired with a CO tip. (e, f) Line profiles along the paths shown by red and blue arrows, respectively, in (d). The distance between local AFM maxima was used to provide an indication of bond lengths at the molecule core. Scalebars: 5 Å

References:

S1. Sengupta, R.; Nakajima, R.; Haketa, Y.; Maeda, H. Porphyrin AuIII Complexes Bearing Bulky Meso-Substituents as Building Blocks of Stacking-Frustrated Ion-Pairing Assemblies. *Org. Lett*. **2025**, *27*, 7395–7399.

S2. Blum, V.; Gehrke, R.; Hanke, F.; Havu, P., Havu, V.; Ren, X.; Reuter, K.; Scheffler, M. Ab initio molecular simulations with numeric atom-centered orbitals. *Comput. Phys. Commun.* **2009**, *180*, 2175-2196.

S3. Neese, F. The ORCA program system. *Wiley Interdiscip. Rev. Comput. Mol. Sci.* **2012**, *2*, 73-78.

S4. Perdew, J. P.; Burke, K.; Ernzerhof, M. Generalized gradient approximation made simple. *Phys. Rev. Lett.* **1996**, *77*, 3865.

S5. Angeli, C.; Cimiraglia, R.; Evangelisti, S.; Leininger, T.; Malrieu, J. P. Introduction of n-electron valence states for multireference perturbation theory. *J. Chem. Phys.* **2001**, *114*, 10252-10264.

S6. Krejčí, O.; Hapala, P.; Ondráček, M.; Jelínek, P. Principles and simulations of high-resolution STM imaging with a flexible tip apex. *Phys. Rev. B* **2017**, *95*, 045407.

S7. Kumar, M.; Soler-Polo, D.; Lozano, M.; Monino, E.; Veis, L.; Jelinek, P. Multireference Theory of Scanning Tunneling Spectroscopy Beyond One-Electron Molecular Orbitals: Can We Image Molecular Orbitals? *J. Am. Chem. Soc.* **2025**, *147*, 24993–25003.

S8. Ortiz, J. V., Dyson-orbital concepts for description of electrons in molecules. *J. Chem. Phys.* **2020**, 153, 070902.34. Verlhac, B.; Bachellier, N.; Garnier, L.; Ormaza, M.; Abufager, P.; Robles, R.; Bocquet, M.-L.; Ternes, M.; Lorente, N.; Limot, L. Atomic-scale spin sensing with a single molecule at the apex of a scanning tunneling microscope. *Science* **2019**, *366*, 623-627.

S9. Martin, R. L. Natural transition orbitals. *J. Chem. Phys.* **2003**, *118*, 4775-4777.

S10. Solé, A. P.; Kumar, M.; Soler-Polo, D.; Stetsovych, O.; Jelínek, P. Nickelocene spm tip as a molecular spin sensor. *J Phys. Condens. Matter.* **2024**, 37, 095802.

S11. Soler-Polo, D.; Stetsovych, O.; Kumar, M.; Lowe, B.; Barragán, A.; Gao, Z.; Solé, A. P.; Zhao, H.; Pérez-Elvira, E.; Goudappagouda, Écija, D.; Narita, A.; Jelínek, P.; Urgel, J. I., Magnetic ground state discrimination of a Polyradical Nanog-raphene using Nickelocene-Functionalized Tips. arXiv 2025. https://arxiv.org/abs/2504.06853
